# Synthesis, characterization, and anticancer evaluation of 1,3-bistetrahydrofuran-2yl-5-FU as a potential agent for pancreatic cancer

**DOI:** 10.1186/s12885-022-10449-y

**Published:** 2022-12-22

**Authors:** Nkafu Bechem Ndemazie, Andriana Inkoom, Dexter Ebesoh, Raviteja Bulusu, Esther Frimpong, Jose Trevino, Bo Han, Xue Zhu, Edward Agyare

**Affiliations:** 1 College of Pharmacy and Pharmaceutical Sciences, Institute of Public Health, Florida A&M University, 1415 South Martin Luther King Jr Blvd, Tallahassee, FL 32307 USA; 2 Faculty of Health Sciences, University of Buea, Buea, Cameroon; 3 Department of Surgery, Virginia Commonwealth University, Richmond, VA 23298 USA; 4 Department of Surgery, Keck School of Medicine University of South California, Los Angeles, CA 90033 USA

**Keywords:** Pancreatic cancer, Modified fluorouracil, Cytotoxicity, Apoptosis, Immunohistochemistry

## Abstract

The failure of current chemotherapeutic agents for pancreatic cancer (PCa) makes it the most aggressive soft tissue tumor with a 5-year survival of slightly above 10% and is estimated to be the second leading cause of cancer death by 2030.

**Objective:** The main aim was to synthesize, characterize and evaluate the anticancer activity of 1,3-bistetrahydrofuran-2yl-5FU (MFU).

**Methods:** MFU was synthesized by using 5-fluorouracil (5-FU) and tetrahydrofuran acetate, and characterized by nuclear magnetic resonance (NMR), micro-elemental analysis, high-performance liquid chromatography (HPLC), and liquid chromatography with mass spectrophotometry (LC-MS). MFU and Gemcitabine hydrochloride (GemHCl) were tested for antiproliferative activity against MiaPaca-2 and Panc-1 cell lines.

**Results:** The half-minimum inhibitory concentration (IC_50_) of MFU was twice lower than that of GemHCl when used in both cell lines. MiaPaca-2 cells (MFU-IC_50_ = 4.5 ± 1.2 μM vs. GemHCl-IC_50_ = 10.3 ± 1.1 μM); meanwhile similar trend was observed in Panc-1 cells (MFU-IC_50_ = 3.0 ± 1 μM vs. GemHCl-IC_50_ = 6.1 ± 1.03 μM). The MFU and GemHCl effects on 3D spheroids showed a similar trend (IC_50-GemHCl_ = 14.3 ± 1.1 μM vs. IC_50-MFU_ = 7.2 ± 1.1 μM) for MiaPaca-2 cells, and (IC_50-GemHCl_ = 16.3 ± 1.1 μM vs. IC_50-MFU_ = 9.2 ± 1.1 μM) for Panc-1 cells. MFU significantly inhibited clonogenic cell growth, and induced cell death via apoptosis. Cell cycle data showed mean PI for GemHCl (48.5–55.7) twice higher than MFU (24.7 to 27.9) for MiaPaca-2 cells, and similarly to Panc-1 cells. The in-vivo model showed intensely stained EGFR (stained brown) in all control, GemHCl and MFU-treated mice bearing subcutaneous PDX tumors, however, HER2 expression was less stained in MFU-treated tumors compared to GemHCl-treated tumors and controls. Mean tumor volume of MFU-treated mice (361 ± 33.5 mm^3^) was three-fold lower than GemHCl-treated mice (1074 ± 181.2 mm^3^) bearing pancreatic PDX tumors.

**Conclusion:** MFU was synthesized with high purity and may have potential anticancer activity against PCa.

**Supplementary Information:**

The online version contains supplementary material available at 10.1186/s12885-022-10449-y.

## Introduction

PCa is the most aggressive and scourging soft tissue tumor worldwide, with a mean age of onset between the 5th and 6th decades of life in the US [1]. According to the Surveillance, epidemiology, and end results (SEER) report of 2022, it is estimated that slightly over 62,000 new cases of PCa will be diagnosed by the end of 2022 with closed to 50,000 people dying from it. This makes PCa the 3rd most common cause of cancer related deaths in the US [2]. Its current incidence is 13.1 per 100,000 US population with a 5-year survival rate of 11.2%, one of the poorest nationwide according to WHO [3]. Estimates show that PCa will be the second leading cause of cancer-related death by 2030, surpassing breast and colorectal cancers [4]. Even though there have been recent advances in the treatment of PCa, there are limited treatment options for PCa patients due to the systemic instability of some existing drugs. Therefore, the synthesis, and characterization of the novel anticancer compound and thorough preclinical evaluation of strategies against PCa is imperative.

Fluoropyrimidines (FP) are increasingly making up the chemotherapeutic backbone of neoadjuvant, adjuvant, and palliative treatment for PCa [5]. 5-Fluorouracil (5-FU) constitutes a component of the current second-line treatment for early disease and combination therapy for late disease which include a cocktail of leucovorin calcium (Folic acid), fluorouracil, irinotecan hydrochloride and oxaliplatin (FOLFIRINOX) [3, 6, 7]. However, their poor absorption, rapid metabolism, rapid systemic elimination, resistance, and undesirable effects are drawbacks to its success in clinical practice [6].

Although GemHCl is the preferred first-line treatment of PCa singly or in combination with other anticancer drugs [8], 5-FU remain the mainstay of chemotherapy currently used in cases of treatment failure, which is quite common in advanced disease due to resistance to gemcitabine [7]. This resistance is usually as a result of the rapid degradation of GemHCl by cytidine deaminase leading to the use of very high doses of the drug resulting to myelosuppression, gastrointestinal toxicity and pulmonary fibrosis in some gcases [9]. FP are mostly prodrugs with a similar mechanism of action to gemcitabine, that involves their bioconversion into nucleotides which are mis-incorporated into ribonucleic acid (RNA) and deoxyribonucleic acid (DNA) strands resulting in cell death [6, 10, 11]. A significant drawback that has challenged the effective use of these drugs remains its systemic instability due to the rapid metabolism by dihydropyrimidine dehydrogenase in the liver into other non-cytotoxic metabolites, which are quickly excreted into feces and urine [10]. This drawback has significantly reduced the half-life of the drugs, decreasing the concentration of the active compound at the target [12, 13]. We exploit this to synthesis MFU which contains two THF rings forming amide bonds at positions 1 and 3 of the 5-FU backbone. Hypothesize that this conjugation will improve systemic stability, increase half-life and provide a kind of hinderance in the activities of DPD on 5-FU metabolism. All these will increase the bioavailability of MFU and prolong the circulation time as well as half-life in circulation, therefore improving anti-cancer activities or converted to 5-FU and improving anti-cancer activities.

This study successfully synthesized, characterized, and evaluated the invitro anticancer activity of FP derivative, 1,3 bistetrahydrofuran-2-yl-5-FU (MFU) in PCa cell lines. Viability studies conducted in 2D and 3D MiaPaca-2 and Panc-1 models demonstrated significant sensitivity to MFU compared with GemHCl. Further, clonogenic studies revealed a remarkable damage to MFU treated MiaPaCa-2 cells than GemHCl treated ones. Equally, we demonstrated pathway of cell death (apoptosis vs necrosis) and the expression of different cellular proteins using western blot analysis following treatment with MFU. Finally, we analyzed the expression of EGFR, HER2 and VEGFR using immunohistochemistry techniques following intravenous treatment of PDX mouse models bearing human pancreatic cancer tumors.

## Materials and methods

### Materials

5-FluoroUracil (5-FU) was purchased from AK Scientific (Union City, CA). Pancreatic cancer MiaPaCa2 (ATCC® CRL1420™) and Panc-1 (ATCC® CRL1469™) were bought from American Type Culture Collection (ATCC) (Manassas, VA). All other solvents and reagents for analysis were purchased from Sigma Aldrich (St. Louis, MO).

### Synthesis of MFU

**Fig. 1:**
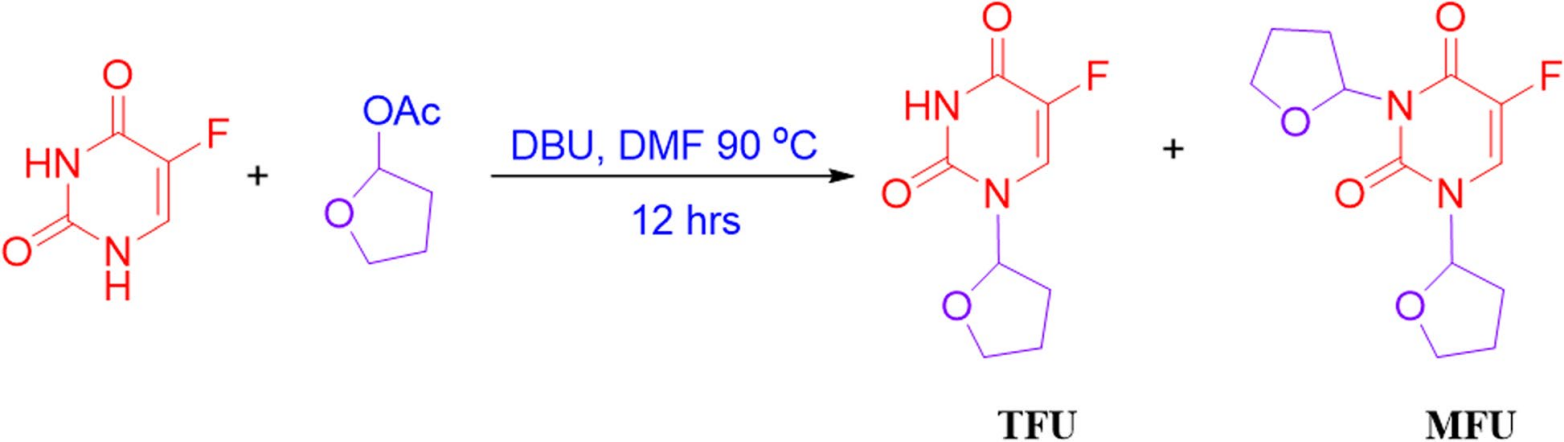
Synthesis of MFU. Synthesis and reaction conditions for the synthesis of MFU, which also yielded TFU (tegafur)

The synthesis of MFU followed a method described by Zasada et al., with some modifications [14]. To a solution of 5-FU (2.1 g, 16 mmol), tetrahydrofuran-2-yl acetate (4.2 g, 32 mmol) in dimethylformamide (DMF, 15 mL) was added to 1,8-diazabicyclo [5.4.0]undec-7-ene (DBU; 5 g, 32 mmol) at room temperature while stirring at 450rpms (Fig. 1). The resulting solution was heated and stirred for 24 h. Most of the DMF was removed in vacuo. The residue was diluted with EtOAc (400 mL) and washed with brine (200 mL). The Organic layer was dried and filtered. The filtrate was concentrated and followed by separation using chromatography on silica gel using EtOAc/Hexane (50%) to obtain MFU. The compound obtained was then crystallized from EtOAc/Hexane as white solid pellets.

The MFU synthesized with the method mentioned earlier was analyzed by proton and carbon-13 Nuclear Magnetic Resonance (NMR) spectroscopy, Micro-elemental analysis, High Performant Liquid Chromatography (HPLC), and High-Resolution Mass Spectrometry (HRMS). The melting point for the solid MFU compound was determined. The purity of MFU was calculated to be 99.6%, as determined by NMR and elemental analysis. Elemental analysis was performed by an independent lab (Atlantic Microlab, Inc., Norcross, GA.). HPLC and HRMS were formed by an independent laboratory to confirm NMR findings.

MFU: ^1^H NMR (CDCl_3,_ 300 MHz): 7.36 (1H, d, *J*_*H-F*_ = 6.0 Hz), 6.60–6.67 (1H, m), 5.97–5.99 (1H, m), 4.29–4.36 (1H, m), 4.20–4.26 (1H, m), 3.93–4.04 (2H, m), 2.44–2.54 (1H, m), 2.33–2.42 (2H, m), 2.20–2.28 (1H, m), 2.03–2.13 (2H, m), 1.86–1.96 (2H, m).

^13^C NMR (CDCl_3_, 151 MHz) δ (a mixture of two rotamers): 157.28 (d, *J* = 25.4 Hz), 157.17 (d, *J* = 25.4 Hz), 148.2, 148.66, 139.94 (d, *J* = 234.3 Hz), 139.87 (d, *J* = 234.3 Hz), 121.75 (d, *J* = 34.2 Hz), 121.58 (d, *J* = 34.2 Hz), 87.97, 87.87, 85.20, 84.99, 70.75, 70.74, 70.27, 70.21, 33.04, 32.92, 28.78, 28.73, 26.51, 26.47, 23.76, 23.70. MP = (114-115 °C), Rf value = 0.36 (100% ethyl-acetate). HPLC (H20/ACN/TEAA (80:15:5), % purity = 100%, RT = 1.85 min) MS (M + H) ^+^ = 270.08. Molecular formula: *C*_*12*_*H*_*15*_*FN*_*2*_*O*_*4,*_ Mol wt: 270.02. Elemental analysis: *Calculated for C*_*12*_*H*_*15*_*FN*_*2*_*O*_*4*_: C 53.33, H 5.59, N 10.37; *Found*: C 53.20, H 5.61, N 10.40.

### Cytotoxicity in 2 and 3 dimensional (2D and 3D) cultures

#### 2D MiaPaca-2 and Panc-1 cells

Both cell lines were incubated in Dulbecco’s modified Eagle medium (DMEM) with high glucose and l-glutamine, supplemented with 10% fetal bovine serum (FBS) and 1% penicillin-streptomycin (PenStrep) [2] and 2.5% 4-(2-hydroxyethyl)-1-piperazineethanesulfonic acid (HEPES). MiaPaca-2 and Panc-1 cells were seeded into 96-well plates at the density of 1 × 10^3^ cells per well in quintuplicate for each drug concentration level and incubated at 5% CO_2_ and a temperature of 37 °C. At 80–85% confluence, both MiaPaca-2, and Panc-1 cells were treated with MFU. A stock solution of MFU was prepared with distilled water and serially diluted with the growth medium to prepare varied concentrations: thus 80, 40, 20, 10, and 5 μM. Both cells were treated with 200 μL of each drug concentration in quintuplicate and incubated for 48 h. At termination, 20 μL of 0.05% resazurin sodium salt (Alamar blue®) was added and incubated at optimum conditions (5% CO_2_, 37 °C) for four h [3]. Fluorimetric analysis was determined at an excitation wavelength of 560/580 nm and emission wavelength of 590/610 nm, and the percent viable cells per concentration was calculated.

#### 3D spheroid of Mia Paca-2 and Panc-1 cells

MiaPaca-2 and Panc-1 cells were plated in 3D formation 96-well plates, Nunclon Sphera® at seeding density of 5.0 × 10^4^ and 2.0 × 10^4^ in 100 μL/well of the growth medium, respectively, the volume of cell suspension in each well plate was then made up to 200 μL with growth medium and centrifuged at 1500rpms and incubated for 24 h to allow for spheroid formation. At treatment, 100 μL of supernatant was replaced with the drug in the growth medium prepared as described under 2D viability studies of Mia Paca-2 and Panc-1 cells and incubated for 48 h. At termination, 50 μL of 0.05% resazurin sodium salt (Alamar blue®) was added to each well and gently dispersed by pipetting and incubated for 4 h. Fluorometric analysis was measured as described above.

#### MFU inhibition of spheroid formation

MiaPaCa-2 and Panc-1 cell suspensions (5.0 × 10^4^ and 2.0 × 10^4^ in 100 μL/well) in treatment media were seeded into the wells of a 96 U Nunclon Sphera plate (Thermo Scientific, Waltham, MA). After 24 h, equivalent amounts of varying concentrations of MFU (0–10 μM) were supplemented to each well of the 96 U plate. The ability of the cells to form viable spheroids was examined at 48 h post-exposure to MFU. The viabilities of the spheroids were determined by staining them with Acridine Orange (AO)/Ethidium Bromide (EB) (5 μg/mL) solution and then capturing fluorescent and bright-field images. The ratios of the fluorescent intensities of AO over EB for the respective MFU concentrations used were computed, and the data was graphed using GraphPad Prism 5.0.

### Stability of MFU

#### Human liver microsome (HLM) stability of MFU

The incubation of MFU (concentration 50 μm) with HLMs was performed in phosphate buffer (pH 7.4) at 37 °C. Then, the incubated samples were shaken for a specified time at 150 rpm. The optimization of conditions for carrying out the process included the incubation time (0–120 min), the concentration of HLM (20 mg/mL), and the reaction buffer consisting of an enzymatic reaction cofactor NADP/NADPH. The blank samples were of the same composition as the test samples; however, they did not contain any of the studied biologically active compounds. The process was terminated with 200 μL of ice-cold methanol. Thereafter, samples were immediately prepared and analyzed with the use of HPLC-UV/MS. All samples were prepared in triplicate. All samples were incubated and treated as described above and as also described in detail as per the manufacturer’s protocol (ThermoFisher Scientific).

#### In vitro stability of MFU

Cells (Mia Paca-2 and Panc-1) were cultured in Dulbecco’s modified Eagle medium (DMEM) with high glucose and l-glutamine, supplemented with 10% fetal bovine serum (FBS) and 1% penicillin-streptomycin (PenStrep) and 2.5% 4-(2-hydroxyethyl)-1-piperazineethanesulfonic acid (HEPES). Additionally, MFU was added to the culture media at the concentrations 12.5 μm, 25 μm, and 50 μm for 24 h and 48 h. Cells were cultured at 37 °C in humidified atmosphere of 5% CO_2_. Cells were plated in T25 culture flask at the density of 1.5 × 10^5^ cells per cm^2^ and grown for 48 h. Then the medium was aspirated at 24 or 48 h. Finally, MFU and potential metabolites were analyzed after filtration from the culture medium.

### MFU induced apoptosis (flow cytometry)

In the morphologic analysis, Annexin V/propidium iodide staining was used as per the manufacturer’s instructions to study the mode of cancer cell death upon exposure to MFU. Mia Paca-2 and Panc-1 cells were seeded into a 25 mL culture flask at 2.0 × 10^4^ cells/mL and left at 37 °C for 24 h to attach. Cells were exposed to MFU (0–10 μM) for 48 h, followed by washing in PBS and labeling with FITC-conjugated Annexin V for 20 min in the dark. Cells were then washed and analyzed using a Becton Dickinson FACSort flow cytometer with CellQuest software (Mansfield, MA).

### Cell cycle analysis

The percentage of cells in the G1 (apoptotic cells), S, and G2/M phases was determined by DNA flow cytometry. In brief, Mia Paca-2 and Panc-1 cells were plated at 1 × 10^5^ cells in a 25 mL flask and allowed to grow until 75–80% confluence. Cells were incubated with different concentrations of GemHCl and MFU (0–10 μM) for 48 h at 37 °C and 5% CO_2_. After treatment, cells were collected by trypsinization, fixed gently in 70% ethanol drop-wisely, and then stored at -20 °C overnight. Then cells were washed in PBS and stained with 0.5 mL PI/RNase for 30 min at room temperature in the dark. Cells were then collected and analyzed using a Becton Dickinson FACSort flow cytometer with CellQuest software (Mansfield, MA).

### Western blot

Panc-1 cells were plated in a T25 flask and cultured for 2 days, respectively, and different concentrations of MFU were added for 48 h. The corresponding cells were collected, washed with PBS, and lysed with RIPA cell lysis buffer to extract the total proteins. The extracted proteins were loaded and subjected to electrophoresis, transferred to a PDVF membrane, the blots were then cut at appropriate molecular sizes prior to hybridization and incubated in the primary antibody overnight. The following day, the primary antibody was recovered, and the corresponding secondary antibody incubated. The immune complexes were detected using enhanced chemiluminescence solution (Biorad) and visualized using the ChemiDoc™ XRS+ imaging system (Bio-Rad).

### Animal studies

#### Ethics statements

Eight-week-old mice were obtained from the Jackson Laboratory (Bar Harbor, ME). The mice were housed in a virus-free environment with indoor light and temperature controlled and provided access to food and water ad libitum for 1 week before treatment started. All procedures with mice were in strict accordance with the National Institutes of Health Guide for the care and Use of Laboratory Animals and the Animal Research Reporting of In Vivo Experiments (ARRIVE) guidelines. This was approved by the Florida A&M University Animal Care and Use Committee.

#### Tumor transplantation

Tumor tissue was surgically implanted in the left flank of immune-compromised mice as previously described [15]. A viable portion of resected tissue was isolated immediately following resection of primary PCa specimens to minimize critical ischemia time. The PCa tissue was then implanted subcutaneously into 8-week-old mice (*n* = 12). Xenografts were allowed to grow to a maximum of 1.5 cm before implantation to the flank of the new host.

#### Tumor efficacy studies

In this study, mice bearing surgically implanted tumors were randomized into groups as control, GemHCl and MFU (*n* = 5/group) once tumor volumes became palpable and reached a range of 70–100 mm [3]. Baseline tumor volumes were established, and dosing initiation began with intravenous administration of 40 mg/kg GemHCl and MFU (with Gem equivalent doses) twice weekly for 6 weeks. Tumor measurements were performed every other day. Tumor volumes were measured using calipers and calculated using the following equation: V = (L*(W)^2^)/2, where V is volume (mm^3^), W (width) is the smaller of two perpendicular tumor axes and the value L (length) is the larger of two perpendicular axes. Tumor growth volumes were calculated for each treatment group.

#### Euthanization

Carbon dioxide (CO_2_) flow to the chamber was adjusted to 3 l per minute for 2 to 3 minutes and observed each mouse for lack of respiration and faded eye color. The CO_2_ flow was maintained for a minimum of 1 minute after respiration was.

ceased and followed by decapitation with scissors. The tumors were then incised and prepared for immunohistochemistry studies.

### Immunohistochemistry (IHC) analysis

Tumor samples excised from the mice, washed with PBS, placed in 10% buffered formalin for 24 h, and transferred to 70% ethanol for histopathological analysis. Immunohistochemistry was performed by Histowiz Inc. (histowiz.com) using a Standard Operating Procedure and fully automated workflow. Samples were processed, embedded in paraffin, and sectioned at 4μm [16]. Immunohistochemistry was performed on a Bond Rx autostainer (Leica Biosystems) with enzyme treatment (1:1000) using standard protocols. Antibodies used were rat monoclonal F4/80 primary antibody (eBioscience, 14–4801, and 1:200) and rabbit anti-rat secondary (Vector, 1:100) [17–19]. Bond Polymer Refine Detection (Leica Biosystems) was used according to the manufacturer’s protocol. After staining, sections were dehydrated, and film cover slipped using a Tissue-Tek Prisma and Cover-slipper (Sakura). Whole slide scanning (40×) was performed on an Aperio AT2 (Leica Biosystems).

### Statistical analysis

All results were presented in the form of means ± SEM. The difference between GemHCl and MFU treatment groups was analyzed using ANOVA, and where necessary, significance was considered for *p* values < 0.05. All experiments were performed in at least triplicates, and analysis was done using GraphPad Prism 5.0 software (GraphPad Software, Inc., San Diego, CA). When necessary, results were presented in simple tables, graphs, and bar charts.

## Results

### Synthesis and characterization of MFU

#### ^1^H and ^13^C NMR analysis

We successfully synthesized MFU (Fig. 1) in good yields and characterized the structure using ^1^H and ^13^C NMR (Supplementary Fig. S1 & S2). Characteristic MFU ^1^H NMR peaks were 1.02 ppm (−C-N on 2′) and 1.04 ppm (−C-N on 2″), representing the amide linkage contributed by the tetrahydrofuran acetate on the 5-FU cyclic structure. The introduced tertiary amine carbonyl carbon in MFU displays a characteristic C-13 NMR peak at 87.87–87.97 ppm and 84.98–85.19 ppm for 2′ and 2″ carbonyl carbon on MFU, respectively. These peaks confirmed the successful conjugation of tetrahydrofuran acetate on 5-FU.

### Micro-elemental analysis

The elemental analysis of MFU is presented in Table 1. It indicated that there are 53.57% of carbon, 5.64% of hydrogen and 10.44% nitrogen in the MFU compound compared with the theoretical values of 53.33% carbon, 5.59% hydrogen and 10.37% nitrogen. This data exhibited a minimum yield of 99.76% confirming high purity (Table 1 and Supplementary Fig. S3).

**Table 1:** Micro-elemental analysis

Element	Theory	Found	Difference	%Purity
**Carbon**	53.33	53.57	0.24	99.76
**Hydrogen**	5.59	5.64	0.05	99.95
**Nitrogen**	10.37	10.44	0.07	99.93

### HPLC and mass spectrometry

The HPLC purity for MFU was greater than 99.6% (Supplementary Fig. S4), indicative of a pure compound synthesized. The retention time was 1.85 minutes. For HRMS studies, the highest intensity was at a mass to charge ratio (m/z ratio) of 270.08, corresponding to the molecular weight of MFU (Supplementary Fig. S5).

### Cytotoxic effect of MFU on MiaPaCa-2 and Panc-1 cells

**Fig. 2:**
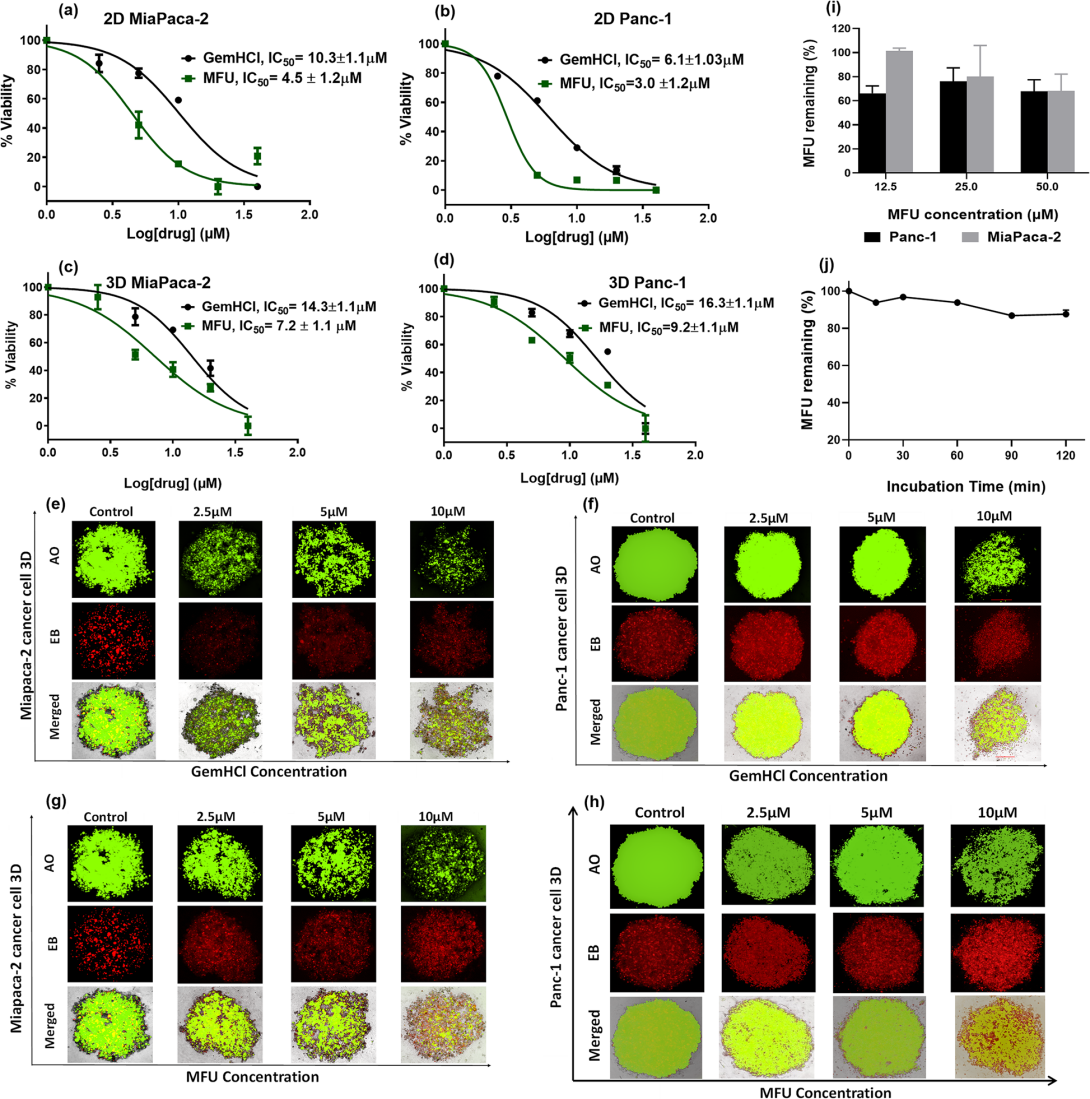
Cytotoxic activity of GemHCl and MFU on MiaPaCa-2 and Panc-1 cells after 48 h incubation and stability studies of MFU. (**a**) and (**b**) 2-D cultured cells showing GemHCl and MFU-treated MiaPaCa-2 and Panc-1 cells, respectively; (**c**) and (**d**) 3-D cultured cells) showing GemHCl and MFU-treated MiaPaCa-2 and Panc-1 cells, respectively. Fluorescent images, (**e**) and (**f**) showing 3-D organoids of MiaPaca-2 and Panc-1 cells treated with GemHCl and (**g**) and (**h**) showing 3-D organoids of MiaPaca-2 and Panc-1 cells treated with MFU. (**i**) Shows percent of MFU remaining after treatment of MiaPaca-2 and Panc-1 cells over 24 h; (**j**) showing percent of intact MFU remaining after exposure to human liver microsomes over 2 h

The cytotoxic activity of MFU was evaluated in comparison to the parent drug GemHCl (first-line anticancer drug in clinical use) in MiaPaCa-2 and Panc-1 cells via 2D culture and 3D spheroids using the Alamar blue assay. As shown in Fig. 2(a) and Table 1, MFU demonstrated significant cytotoxic activity against 2D MiaPaCa-2 culture with an IC_50_ value of 4.5 ± 1.2 μM, compared to GemHCl after 48 h treatment with an almost two-and-a-half-fold increase for GemHCl (10.3 ± 1.1 μM) treated groups, respectively. While IC_50_ values were expectedly higher in the treated 3D MiaPaCa-2 culture compared with that of 2D MiaPaCa-2 culture (Fig. 2a and c) and Table 2), MFU treated 3D MiaPaCa-2 culture (IC_50_ = 7.2 ± 1.1 μM) showed lower IC_50_ values compared with GemHCl treated 3D MiaPaCa-2 culture (IC_50_ = 14.3 ± 1.1 μM). A similar trend was observed for studies performed with Panc-1 cells with similar findings in 2D (reduced IC_50_ values) and 3D (increased IC_50_ values compared to 2D findings) cultures (Fig.2(b and d)), respectively.

**Table 2:** Comparing the IC_50_ values of GemHCl and MFU for Panc-1 and MiaPaCa-2 treated cells in 2-D and 3-D models

Cell lines	GemHCl ± SD (μM)	MFU ± SD (μM)	***P***-Value
MiaPaca-2 (2D)	10.3 ± 1.1	4.5 ± 1.2	< 0.01
Panc-1 (2D)	6.1 ± 1.03	3.0 ± 1.2	0.002
MiaPaca-2 (3D)	14.3 ± 1.1	7.2 ± 1.1	< 0.01
Panc-1 (3D)	16.3 ± 1.1	9.2 ± 1.1	< 0.01

### MFU prevents spheroid formation on MiaPaCa-2 and Panc-1 cells

To better observe drug response characteristics in a system that better simulates the in vivo situation of tumors, the effect of MFU was studied using a 3D spheroids culture. As shown in Fig. 2(e-h), MFU induced a significant disruption of already established spheroids generated with either MiaPaca-2 or Panc-1 cells when compared to GemHCl treated MiaPaca-2 or Panc-1 cells. Further, significant increase in spheroidal disruption or increase in spheroidal diameter was observed as the GemHCl or MFU concentrations increases in the merged images. These merged images indicate more death cells at higher concentrations of MFU compared to that of GemHCl in both cell lines. The disruption of spheroid shape and cell death occurred at relatively higher concentrations of MFU (5–10 μM). The IC_50_ values also demonstrate better cell death in 3D by MFU when compared to GemHCl (7.2 ± 1.1 μM and 9.2 ± 1.1 μM for MiaPaca-2 and Panc-1 cell lines, respectively, Table 2 and Fig. 2(c and d)).

### MFU stability

#### In vitro stability of MFU using PCa cells

Following treatment of MiaPaca-2 and Panc-1 cells with different doses of MFU (12.5 mM to 50 mM) over 24 h, we realized that more than half the initial concentration of MFU in all cases was intact after 24 h treatment in both cell lines (Fig. 2i). There was no statistically significant difference in the initial concentration of MFU after 24 h in both cell lines. This could mean that MFU is quite stable in vitro, and its activities seems to be longer than that GemHCl whose concentration falls to less than half initial concentration between 32 to 94 minutes as documented in literature [20–22].

#### In vitro metabolic stability of MFU using human liver microsomes

In vitro metabolism of MFU in human liver microsomes was investigated using HPLC and the findings shown in Fig. 2j. Percent unchanged or intact MFU was plotted against different time points and the HPLC analysis of percent MFU remaining was greater than 80% after 2 h. However, similar study performed to determine the metabolic stability of GemHCl using human liver microsomes revealed less than 50% of intact GemHCl remaining after 2 h [23]. This implies that MFU may have better metabolic stability compared with GemHCl as reported in literature [24, 25].

### MFU induced apoptosis in MiaPaca-2 and Panc-1 cells

**Fig. 3:**
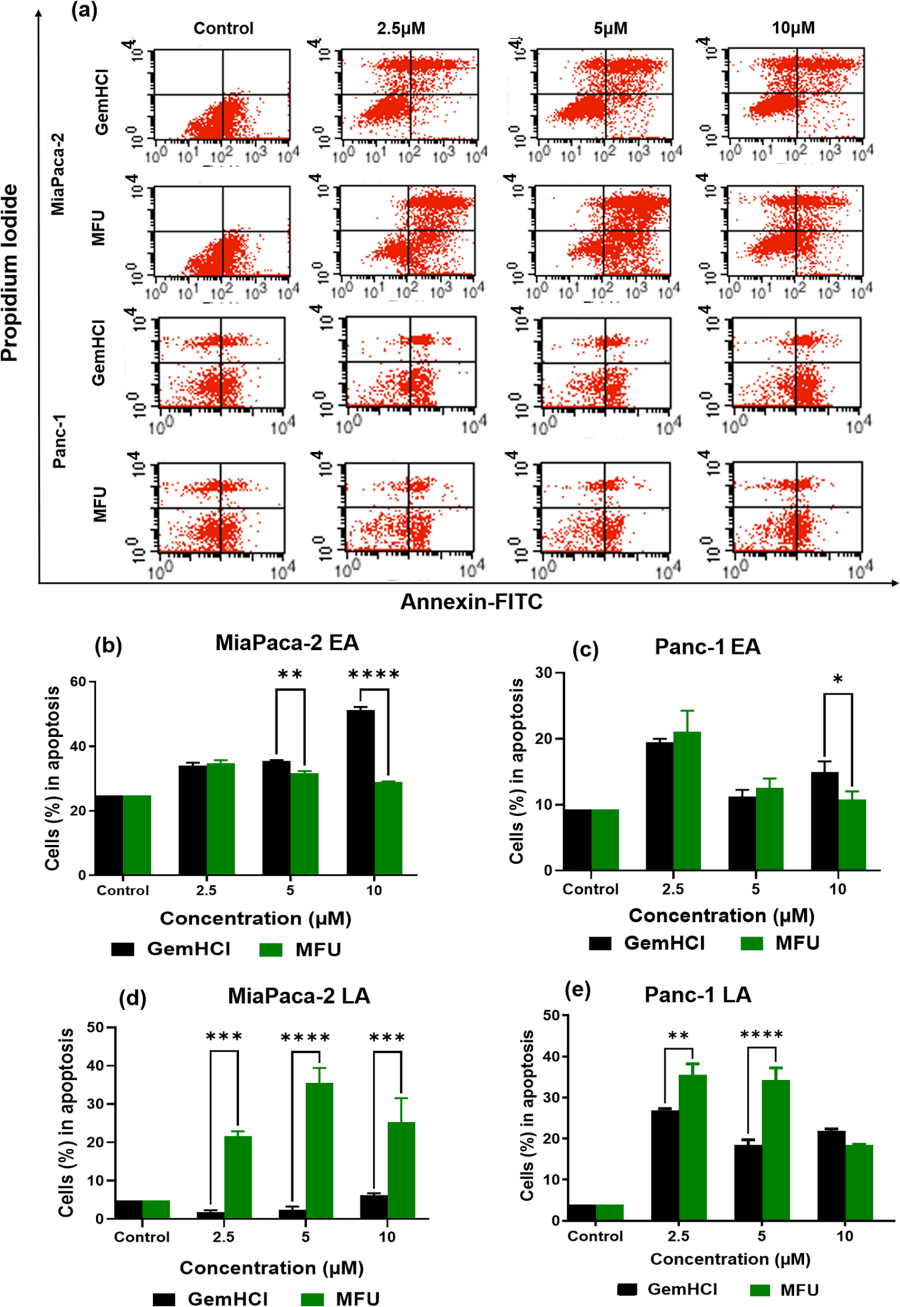
Flow cytometry images showing apoptosis studies at different stages following treatment with GemHCl and MFU using MiaPaca-2 and Panc-1 cells (**a**). (**b**) and (**c**) Miapaca-2 and Panc-1 cells showing early apoptosis, respectively; (**d**) and (**e**) MiaPaca-2 and Panc-1 cells at late apoptosis respectively

The effect of MFU on inducing cell apoptosis was analyzed and shown in Fig. 3. MiaPaca-2 and Panc-1 cells were incubated with MFU at different concentrations for 48 h. Annexin V-FITC/PI staining was carried out, and the percentage of apoptotic cells was further determined using flow cytometry. The results showed that the MFU induced the apoptosis in a dose-dependent manner in both MiaPaca-2 and Panc-1 cells, which were comparable to GemHCl. As shown in Fig. 3a, in MiaPaca-2 cells, the apoptosis rates at concentrations 2.5 μM, 5 μM, and 10 μM of MFU followed an inverse relationship with percentage of cells at early apoptosis which were 35, 31.6, and 29%, respectively (Fig. 3b). In Panc-1, the induced apoptosis rates at concentrations 2.5 μM, 5 μM, and 10 μM of MFU in early apoptosis were 35, 34, and 19%, respectively (Fig. 3c). There percentage number of cells arrested at early apoptosis was similar in both GemHCl and MFU treated cells in both cell lines while the there was a significant increase in the number of cells arrested at late apoptosis when treated with MFU compared to GemHCl (Fig. 3(b-e)). However, at the late apoptosis stage, there was a direct relationship between percentage number of cells and concentration of MFU and GemHCl respectively over the time of the study. These results suggested that cells in EA could be reverted to live cells by cellular repair mechanism, while those in late apoptosis would hardly revert to live cells [26]. This suggest that MFU seems to be superior to GemHCl as more cells treated with MFU were at LA compared to GemHCl treated cells in which more cells were at EA after 48 h of exposure.

### Cell cycle analysis

**Fig. 4:**
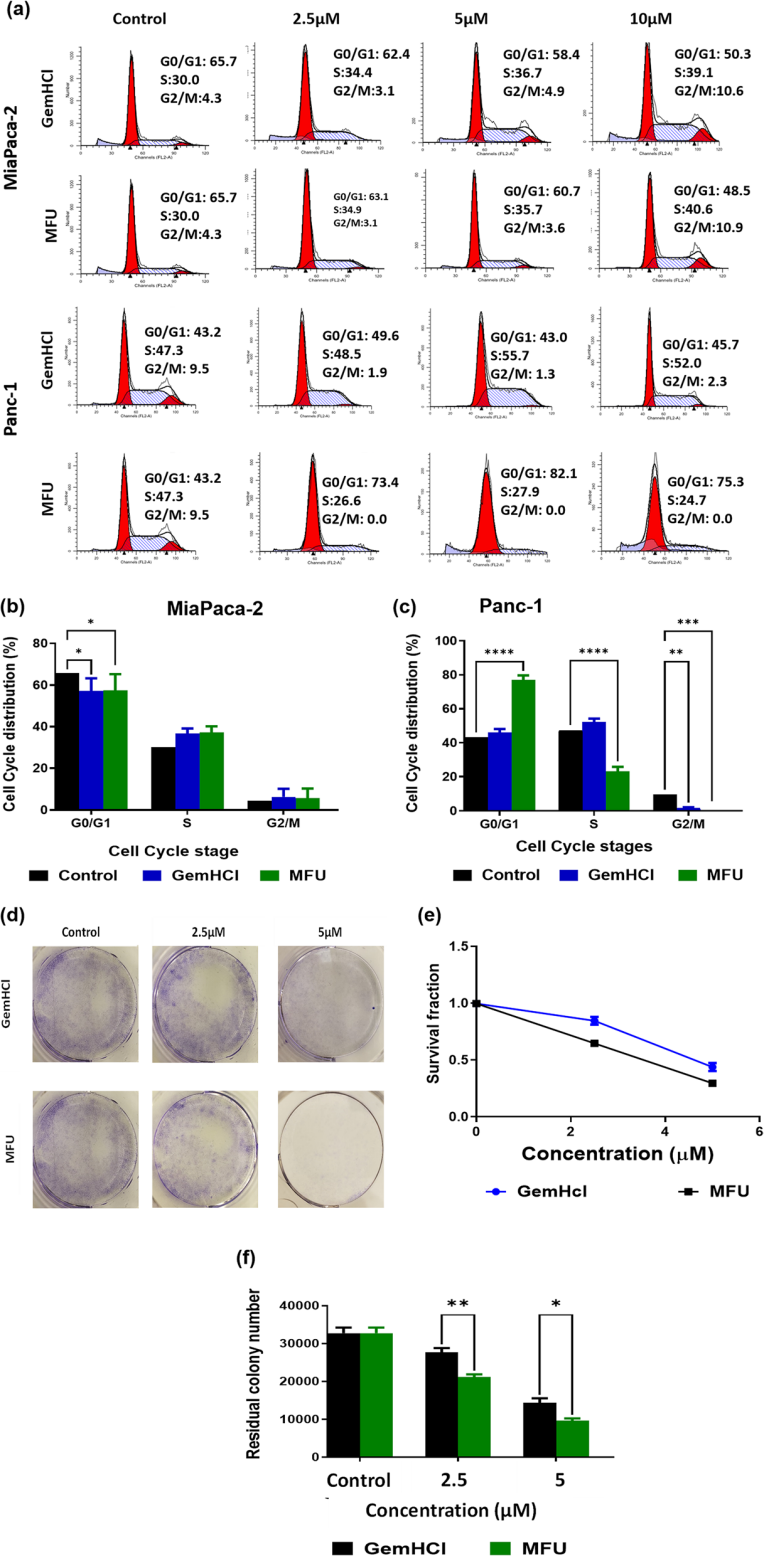
Cell cycle studies for MiaPaca-2 and Panc-1 cells following treatment with GemHCl and MFU and clonogenic studies using MiaPaca-2 cells. (**a**) Flow cytometry images of MaiPaca-2 and Panc-1 cells arrested at different phases of the cell cycle. (**b**) and (**c**) MiaPaca-2 and Panc-1 cells showing percentage of cells at different phases of the cell cycle, respectively. (**d**) photo images captured MiaPaca-2 cells after 2 weeks of growth following 48 h of incubation with GemHCl and MFU; (**e**) and (**f**) survival fraction and residual colony numbers respectively after 2 weeks growth following 48 h of incubation with GemHCl and MFU

To explore what stage of the cell cycle contributed to the decrease in cell proliferation rate, we examined the cell cycles of MiaPaca-2 and panc-1 cells. Cells were cultured in six-well plates divided into four groups, corresponding to 0, 2.5, 5, and 10 μM concentrations described in the method section. The experimental results show that MFU significantly slowed the cell cycle progression. The percentage of cells entering the S and G2/M phases was significantly lower than control (Fig. 4a), whereas the GemHCl showed similar differences in the cell cycle distribution. The mean PI (percentage of the S + G2/M phases), an index reflecting the proliferation characteristic of cells, was also evaluated. The PI of MFU (24.7 to 27.9) was significantly lower than those of the control group (57.3), GemHCl (48.5 to 55.7) for Panc-1 cells, and similar for MiaPaca-2 (Fig. 4b&4c). These data suggest that MFU prolonged the G1 phase of the cell cycle by blocking cell transition from the G1 to S and G2 phases.

### Clonogenic cell culture

We examined the cytotoxic effect of MFU in colony formation assays. We used these assays to assess the ability of the MFU to prevent tumor relapse after treatment. As shown in Fig. 4d, MFU induced concentration-dependent inhibition of clonogenic cell survival on MiaPaca-2 cell lines. Survival fraction in terms of residual colony number (Fig. 4e). There was a significant reduction in colonies when treated with MFU at 2.5 μM and 5 μM concentrations. The inhibition of colony numbers was concentration-dependent with significantly limited colony formation on exposure to both 2.5 μM and 5 μM MFU in MiaPaca-2 cells. These results are consistent with the effects of MFU on cell viability (Fig. 2 (a-d)). In addition, the number of residual colonies were significantly lover in MFU treated cells when compared to GemHCl (Fig. 4f) suggesting the possibility of MFU having a longer half-life and capable of inhibiting cancer cells growth after treatment (relapse).

### MFU induced-apoptosis involved caspase-9 activation and antiproliferative proteins

**Fig. 5:**
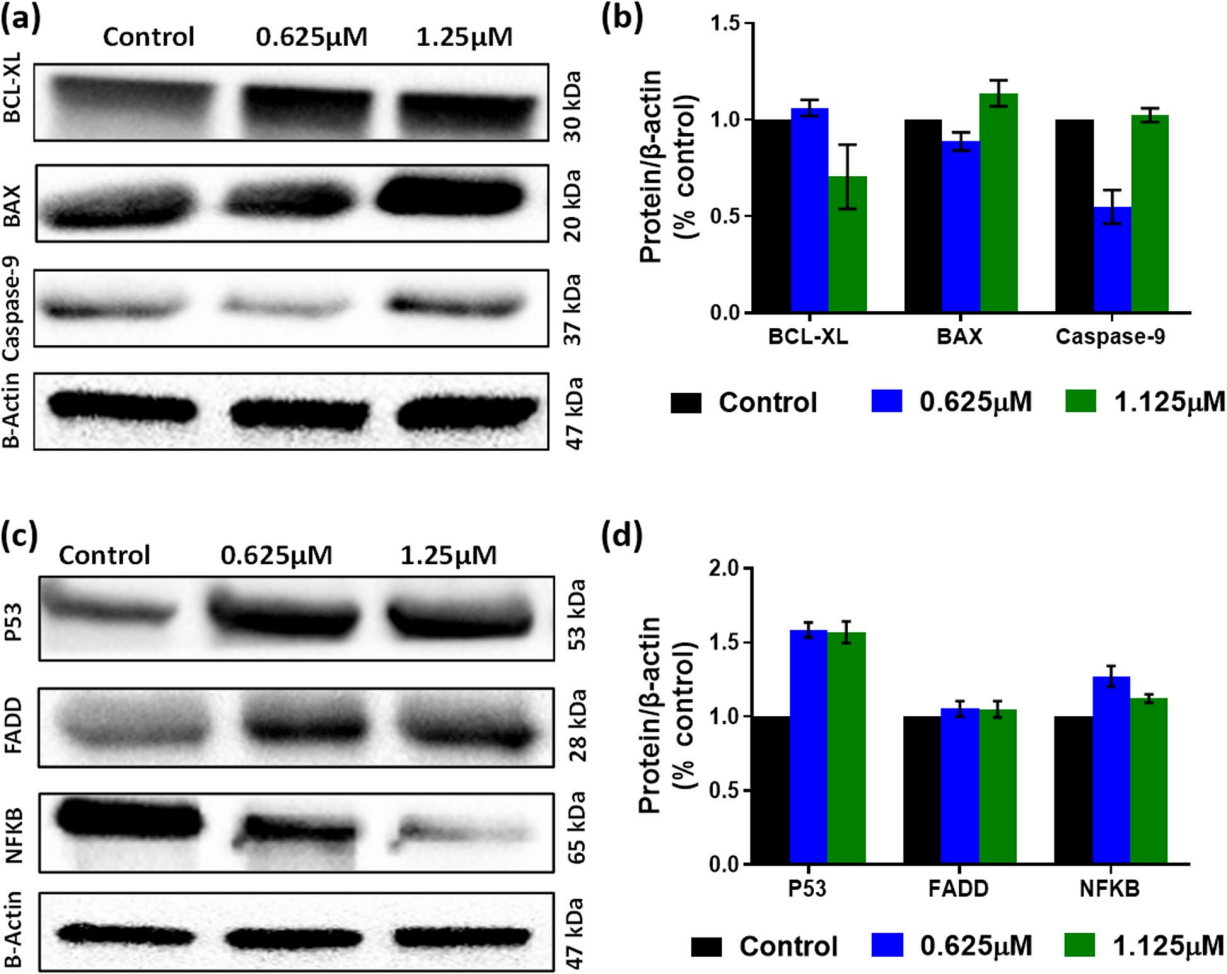
Protein bands after treatment with MFU after treatment with GemHCl and MFU. For protein band expression, the blots were cut prior to hybridization with primary antibodies during the blotting. (**a**) Expression of apoptotic protein including Caspase-9, (**b**) quantitative protein bands expressed, (**c**) bands expressing cell cycle protein, (**d**) quantity of cell cycle proteins expressed

Apoptosis is the process of programmed cell death, and this process mobilizes different proteins via different pathways. These could be caspase-dependent or caspase-independent, with the former involving the activation of caspase-8 or caspase-9 for extrinsic and intrinsic pathways, respectively. We investigated the molecular mechanism underlying the MFU-induced apoptosis by comparing the expression of various intrinsic pathway pro-apoptotic and anti-apoptotic proteins after inducing concentration-dependent cell death with MFU. We observed a concentration-related increase in the expression of caspase-9 and Bax. At the same time, there was an inverse relationship in BCL-XL expression to the concentration of MFU after 48 h (Fig. 5(a and b)). We also investigated the expression of Fas associated death domain (FADD) for the extrinsic pathway which showed a concentration dependent increase in expression. While NF-KB decreased with increasing concentration of MFU and P53 had no significant difference in expression when the concentration of MFU was increase but however was highly expressed in MFU treated cells compared to controls. (Fig. 5(c and d)).

### Tumor efficacy studies

**Fig. 6:**
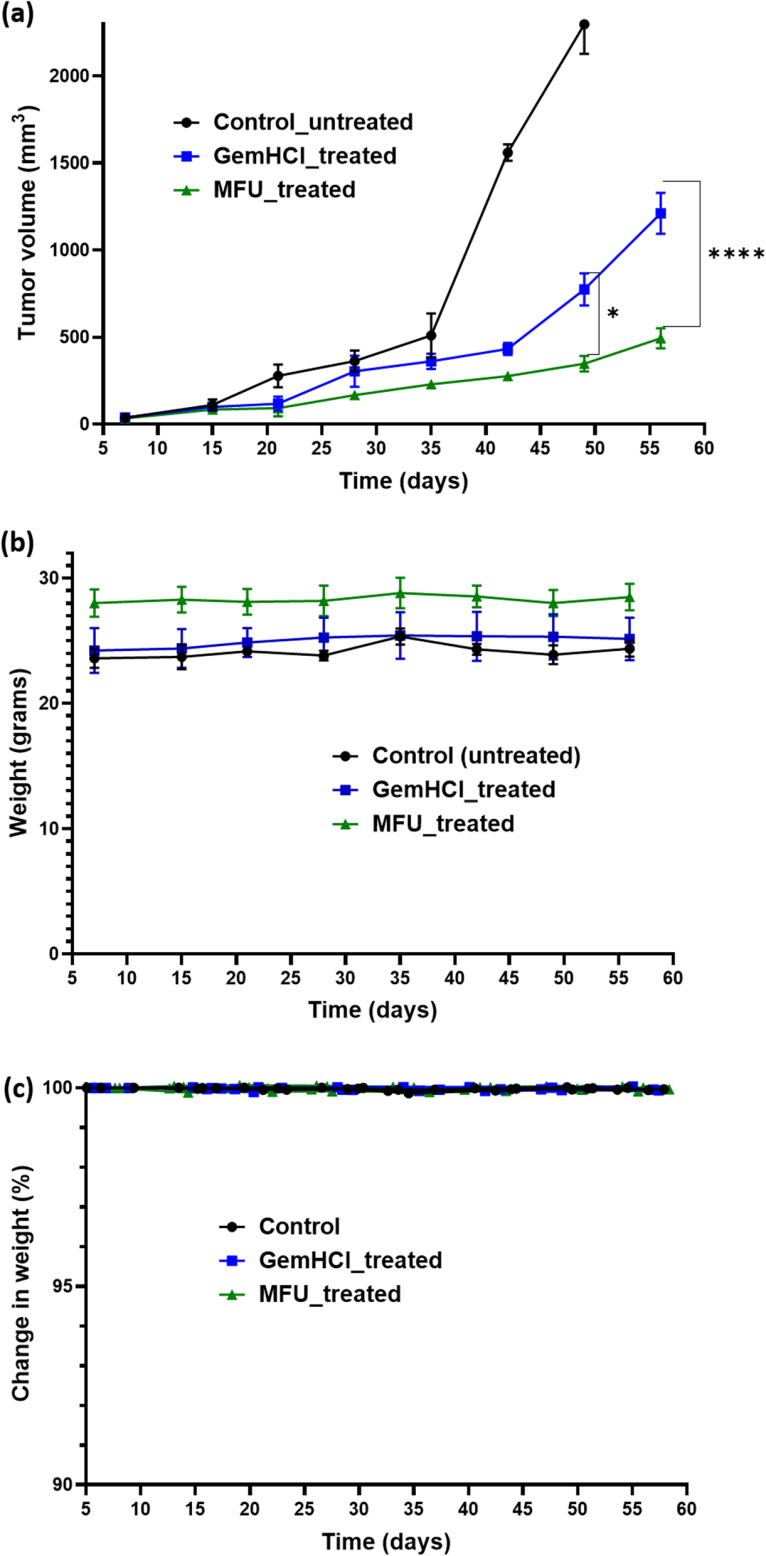
In-vivo efficacy of MFU in PDX mouse model and chronic toxicity. (**a**) Tumor growth curves of GemHCl and MFU treated mice bearing pancreatic PDX tumor and (**b**) body weight during treatment. (**e**) Percent change in the body weight of mice over the study period. Asterisks represents level of significance between control and treatment group (**p < 0.05, ***p < 0.001). All data represents mean ± SD, (*n* = 4/group)

Mice bearing pancreatic cancer PDX tumor were treated over a period of 6 weeks. In the MFU treated group, a significant tumor growth suppression was observed compared with the control and GemHCl treated groups (Fig. 6a). Tumor suppression was noticed up to 5 weeks after which GemHCl-treated group noticed a geometric increase while the MFU-treated group maintained same tumor volume for the rest of study period. The mean tumor volume of the control group was extremely large, the mean tumor volume of MFU-treated group (361 ± 33.5 mm^3^) exhibited significantly lower tumor growth compared with the mean tumor volume of GemHCl-treated group (1074 ± 181.2 mm^3^) especially on week 7 and 8. Figure 6b shows percentage change in the weight of the mice over the study period which is a representation of the toxicity of the drugs/compound on the animal. There is an insignificant percentage change in weight of mice between 99.80 to 100.1% during the study period which is less than 10% recommended to be due to toxicity of a drug [27].

### Immunohistochemistry

**Fig. 7:**
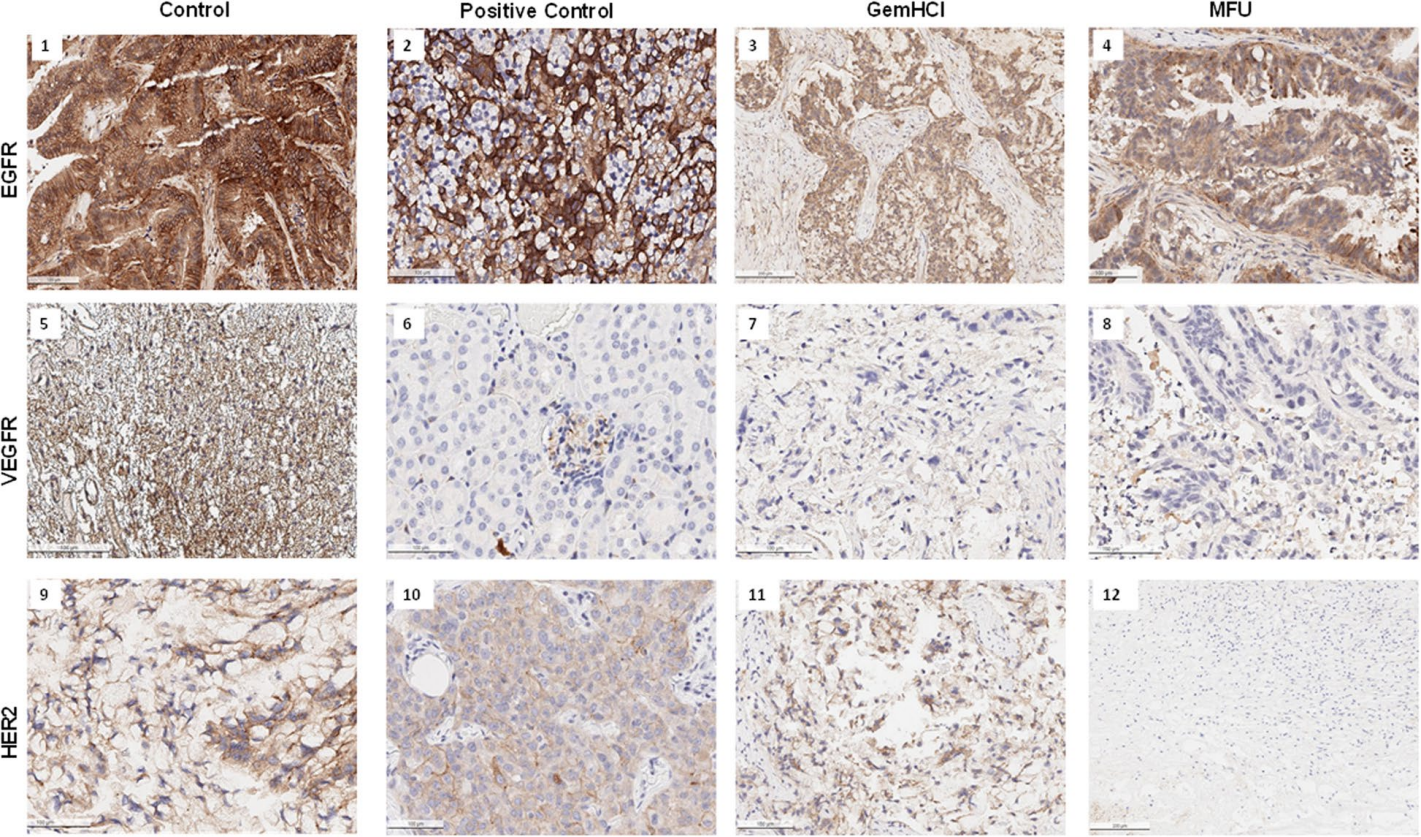
Immunohistochemistry of tumors after treatment. Expression of EGFR, VEGFR and HER2 after treatment of PDX Pca mice bearing tumors with GemHCl and MFU with untreated control

Tyrosine kinase receptors (EGFR, VEGFR, and HER2) are highly expressed in many solid tumors, including PCa. These receptors are reported to be associated with cancer cell proliferation, survival, migration, and differentiation and a factor of poor prognosis [28]. In this study, we evaluated the impact of the novel compound MFU on the expression of EGFR, HER2, and VEGFR in patient-derived pancreatic tumor tissues implanted in mice. The immunohistochemical staining of EGFR, VEGFR, and HER2 is shown in Fig. 7, with brown regions corresponding to highly positive staining. Images 1, 2, 3 and 4 show the expression of EGFR with an intense expression on control, and positive controls, GemHCl and MFU-treated mice tumors (intense brown color) (Fig. 7 images [1–4]). Images [5–8] shows VEGFR expression which is strongly stained in controls (intensely brown colored), positive controls show moderate expression of VEGFR (blue to brown color), but weakly expressed (bluish color) for GemHCl and MFU treated cells. Finally, HER2 seems to be strongly stained in control, positive control and GemHCl treated tumor compared to MFU treated tumors, this suggest that MFU is capable of inhibiting HER2 expression images (9–12-) thus inhibiting tumor growth and survival.

## Discussion

GemHCl and fluoropyrimidines have been widely documented and demonstrated to suppress tumor growth effectively and significantly in PCa cell lines [29–34]. Their use has resulted in severe systemic toxicity because of their short life and higher doses to attain therapeutic effects [35, 36]. However, their importance in the treatment of PCa cannot be overemphasized as they are still the first line therapy in clinical practice. This research study underscores the importance and benefits of a synthesized 1,3-bistetrahydrofuran-2yl-5-FU (MFU) for both in vitro and preliminary in vivo studies using MiaPaca-2 and Panc-1 cells. Although many studies have demonstrated the effectiveness of analogs of Gemcitabine [29, 30], we synthesized a compound that has proven to be more effective than existing drugs.

MFU was successfully synthesized and characterized using NMR, elemental analysis, HPLC, and HRMS (Supplementary Fig. S1-S5). The purity of MFU was more than 99.6%, suggesting that the synthesized compound used in vitro and in vivo in this study was pure and free of unwanted contaminants. As shown in Fig. 2, IC_50_ values of MFU were significantly lower than corresponding values for GemHCl, implying that MFU has higher antiproliferative activity against 2D and 3D MiaPaca-2 and Panc-1 cultures.

The uniqueness of MFU that might have been attributed to its high anticancer activity was probably due to the modification of the polar nature of fluoropyrimidine backbone leading to moderately high lipophilicity. In addition, extensive metabolism of GemHCl and fluoropyrimidines by intracytoplasmic enzymes such as, cytidine deaminase and dihydropyrimidine dehydrogenase respectively, is responsible for their resistance [13]. It has been demonstrated that the amine bond in MFU can be cleaved in cancer cells, releasing the tetrahydrofuran group, thus making the compound behave like 5-fluorouracil (5-FU) [37]. This suggests that MFU is converted to 5-FU in vitro. The released 5-FU is actively converted to its metabolite fluorodeoxyuracil triphosphate (FdUTP), which competitively inhibits thymidine synthetase (TS) and blocks DNA synthesis through the appropriate pathway involved in intracellular phosphorylation [38]. However, this does not exclude the possibility of MFU behaving like a new drug with other mechanism of actions yet to be determined.

Also, some studies have reported that one of the main issues with GemHCl is that it relies much on nucleoside transporters (hENT1) for its delivery and accumulation in PCa cells and thus under-expression of these receptors contributes to the resistance of GemHCl to PCa [39]. The high lipophilicity of MFU compared to GemHCl makes them a better compound to be successfully transported intracellularly for anticancer activity. Although MFU can also be hydrolyzed intracellularly prior to getting to the compartment for phosphorylation, this may limit its efficacy due to deamination prior to phosphorylation. Nevertheless, this current study tentatively shows MFU having an extensive cellular uptake and efficacy compared to GemHCl due to higher transmembrane permeability secondary to its lipophilicity.

In addition, results of cell-induced death or apoptotic studies further support the assertion that MFU exhibits strong cytotoxic effects in 2D and 3D spheroids compared to GemHCl. To further evaluate the stability of MFU, we treated Miapaca-2 and Panc-1 cells at different concentrations of MFU over 24 h and used HPLC to determine the concentration of unchanged MFU. Human liver microsomes were used to determine the systemic stability of MFU. We found that MFU was very stable both in vitro and in human liver microsomal studies with more than 80% remaining intact after 2 h, suggestive that it is more stable compared to GemHCl as reported in literature [8, 20–22, 24, 25].

To further assess the anti-survival effects of MFU, we examined the viability panel of PCa cells grown as monolayers and spheroids. In these assays, we observed a dose-dependent inhibition of the viability of PCa cell lines. To gauge the clinical potential of MFU, we compared their efficacy to GemHCl currently in clinical use for the treatment of PCa: MFU induced cell death at concentrations (IC_50_) that were twice lower than GemHCl in both monolayer and spheroid treated PCa cell lines used. The ability of MFU to inhibit PCa Cell lines is not surprising as MFU was designed to mimic activities of other existing fluoropyrimidines like Fluorouracil which is a component of one of the currently used regimens in clinical practice (FOLFIRINOX) [40]. The tetrahydrofuran moiety may disrupt the interaction between DPD and the fluoropyrimidine backbone, thereby giving room for the intracellular conversion of MFU to FdUTP and further performing its inhibition of Thymidine Sythetase (TS) and blocking DNA synthesis. Notwithstanding, MFU could be inhibiting other pathways in cellular metabolism resulting to novel mechanism of action different from what we hypothesize.

The previous observation that MFU inhibits the viability of PCa cells led us to investigate the mechanism by which the MFU causes the death of these cells. Programmed cell death (apoptosis) is orchestrated by a series of committed signaling events that ultimately lead to the activation of proteases known as Caspases. After conducting apoptotic and necrosis studies, it was evident that MFU causes cell death through apoptosis. We further investigated the expression of pro-apoptotic and anti-apoptotic protein expression in the cells after exposing them to MFU over 48 h. We investigated the expression of BCL-XL, BAX, and Caspase-9; we obtained a decreasing trend in BCL-XL expression, while an increasing trend was observed in the expression of BAX and Caspase-9, indicating apoptosis was the mechanism of cell death through the activation of mitochondria (intrinsic) pathway of apoptosis. Furthermore, the expression of p53 tumor suppressor protein was assessed, showing an increase in the expression of p53 compared to control. We observed dose-independent regulation of uncontrolled cell growth by the activation of p53 in treated cells compared to control. We also observed a dose dependent increase in the expression of FADD in MFU treated cells, this corresponds with literature as 5-FU increases the expression of FADD in the extrinsic pathway of apoptosis [41]. Finally, NFKB was assessed; these are linked to the anti-inflammatory effects of of MFU to PCa cells., we observed dose-dependent increases in expression levels compared to control or untreated cells. This could explain the observation of reduced IC_50_ in spheroids compared to GemHCl and clonogenic studies performed.

In addition to inducing apoptosis, anti-inflammatory, suppressing survival, and resistance to treatment, effective cancer agents, must have chemo-preventive properties to avoid tumor relapse. This was equally investigated in assays where we pre-treated PCa cells with MFU and GemHCl and then examined their survival and growth to form colonies. In these assays, MFU markedly suppressed cell survival and growth compared to GemHCl, indicating that MFU is a potential chemo-preventive agent.

Anti-tumor activity of MFU after the 40th day exhibited a significant inhibition of tumor growth compared with GemHCl. There was a significant decrease in tumor volume for MFU-treated mice (361 ± 33.5 mm^3^) compared to GemHCl-treated mice (1074 ± 181.2 mm^3^) at the end of the studies (*P*-value< 0.001). This extraordinary tumor efficacy of MFU could be explained by two major factors: i) absence of free NH_2_-group on MFU which rendered the DPD enzyme ineffective to metabolize MFU. This event most likely allowed for prolong circulation, increased bioavailability and improved therapeutic efficacy of MFU and ii) conjugation of THF to 5-FU may have imparted some degree of lipophilicity to MFU which may have facilitated its delivery to cancer cells. EGFR, VEGF, and HER2 are tyrosine kinase receptors highly expressed in several solid tumors, including PCa [42, 43]. These receptors are frequently reported to harbor aberrant activities that lead to the proliferation, survival, resistance, and differentiation required for PCa pathogenesis. We investigated the effects of GemHCl and MFU on the expressions of these receptors in the PDX mouse model bearing ectopic pancreatic tumor. In this PDX model, high expressions of EGFR and HER2 were observed after IHC staining tumor of GemHCl treated groups. In MFU treated groups, we observed weak staining with HER2 suggesting that MFU may be effectively used to treat PCa compared to GemHCl as previously reported in literature [44]. In addition, a significant partner of EGFR, in this case, HER2 has been reported to be overexpressed in numerous human cancers with associated drug resistance [17–19, 45]. This could be the case in our study as the PDX pancreatic tumors were obtained from patients already on treatment for PCa before surgery, which might have stimulated the overexpression resulting in resistance to treatment. Also, while there was no significant difference between control and GemHCl-treated tumors in the expression of EGFR and HER2, there was a significant difference in the expression of EGFR and HER2 in MFU-treated tumors. Signifying that MFU-treated tumors could be better for GemHCl resistant tumors with over expression EGFR and HER2. Finally, there was no significant difference in the expression of VEGFR in both GemHCl, and MFU-treated tumors, however VEGFR was highly expressed in the controls. Based on this, we could suggest that treatment with MFU may have had little to no effect in the expression of VEGFR the same as GemHCl, and this corresponds with literature as PCa is known not to have invasive blood supply (one of the rare tumors with no neovascularization) [46]. However, the observable similarities in VEGFR levels account for the insignificant difference in tumor volumes between mice treated with GemHCl versus MFU as the patients might have received chemotherapy and tumors sensitized.

## Conclusion

MFU was successfully synthesized with a high purity. This study demonstrated that synthesized MFU remarkably suppressed inflammation and cell growth, proliferation, and survival through Caspase-dependent apoptosis. Also, the significant hallmarks of cancer are self-sufficiency in growth signals, insensitivity to anti-growth signals, evasion of apoptosis, limitless replicative potential, induction of angiogenesis, and activation of invasion and metastasis. Our study indicates that MFU inhibits growth, induces apoptosis, improves sensitivity to anti-growth signals, and has limited replicative potentials and metastasis. These results collectively indicate that MFU attenuates four of the six major hallmarks capabilities of cancers and underscores their potential usefulness as an effective agent in anticancer strategies.

## Supplementary Information

**Additional file 1: Figure 1.** ^1^H NMR analysis for MFU: ^1^H NMR (CDCl_3,_ 300 MHz): 7.36 (1H, d, *J*_*H-F*_ = 6.0 Hz), 6.60–6.67 (1H, m), 5.97–5.99 (1H, m), 4.32 (1H, td, *J* = 6.3, 8.4 Hz), 4.23 (1H, dt, *J* = 4.2, 8.7 Hz), 3.93–4.04 (2H, m), 2.44–2.54 (1H, m), 2.33–2.42 (2H, m), 2.20–2.28 (1H, m), 2.03–2.13 (2H, m), 1.86–1.96 (2H, m). **Figure 2.** ^13^C NMR analysis for MFU: ^13^C NMR (CDCl_3_, 151 MHz) δ (a mixture of two rotamers): 157.28 (d, J = 25.4 Hz), 157.17 (d, J = 25.4 Hz), 148.2, 148.66, 139.94 (d, J = 234.3 Hz), 139.87 (d, J = 234.3 Hz), 121.75 (d, J = 34.2 Hz), 121.58 (d, J = 34.2 Hz), 87.97, 87.87, 85.20, 84.99, 70.75, 70.74, 70.27, 70.21, 33.04, 32.92, 28.78, 28.73, 26.51, 26.47, 23.76, 23.70. **Figure 3.** Micro-elemental analysis of MFU showing the percent by mass of the elements carbon, hydrogen and nitrogen in the structure of MFU compared to theoretical estimates. **Figure 4.** HPLC analysis of MFU showing a sharp peak at retention time of 1.85, performed by an independent laboratory using acetonitrile/water at a gradient of 20–90% with triethylammonium acetate (50 mM) additive. **Figure 5.** HRMS analysis of MFU. **Supplement Table 1*.*** Pyrimidine derivatives currently used as anticancer in clinical settings. **Figure 6.** NFKB taking at 1 sec (a), 2 secs (b), 3 secs (c) and 4 secs (d). **Figure 7.** FADD images taken at 1 sec (a), 6 secs (b), 12 secs (c) and 18 secs (d). **Figure 8.** B-actin images taken at 1 sec (a), 2 secs (b), 3 secs (c) and 4 secs (d). **Figure 9.** Images of BAX taken at 1 sec (a), 6 secs (b), 12 secs (c) and 18 secs (d). **Figure 10.** Caspase-9 images taken at 1 sec (a), 6 secs (b), 12 secs (c) and 18 secs (d). **Figure 11.** p53 images taken at 1 sec (a), 3 secs (b), 6 secs (c) and 9 secs (d). **Figure 12.** BCL-XL images taken at 1 sec (a), 3 secs (b), 6 secs (c) and 9 secs (d). **Figure 13.** In vitro studies comparing the efficiency of 5-FU and MFU. a) 2D culture of MiaPaca-2 cells, b) 2D culture of Panc-1 cells, c) 3D culture of Miapaca-2 cells and d) 3D culture of Panc-1 cells.

## Data Availability

Data will be made available upon request by the corresponding.
