# Supplementary material for: Synthesis, characterization, and anticancer evaluation of 1,3-bistetrahydrofuran-2yl-5-FU as a potential agent for pancreatic cancer
Source: BMC Cancer. 2022 Dec 22;22:1345. doi: 10.1186/s12885-022-10449-y (PMC9773620; doi:10.1186/s12885-022-10449-y)
Supplement: Supplementary file 1 — Additional file 1: Figure 1. 1H NMR analysis for MFU: 1H NMR (CDCl3, 300 MHz): 7.36 (1H, d, JH-F = 6.0 Hz), 6.60–6.67 (1H, m), 5.97–5.99 (1H, m), 4.32 (1H, td, J = 6.3, 8.4 Hz), 4.23 (1H, dt, J = 4.2, 8.7 Hz), 3.93–4.04 (2H, m), 2.44–2.54 (1H, m), 2.33–2.42 (2H, m), 2.20–2.28 (1H, m), 2.03–2.13 (2H, m), 1.86–1.96 (2H, m). Figure 2. 13C NMR analysis for MFU: 13C NMR (CDCl3, 151 MHz) δ (a mixture of two rotamers): 157.28 (d, J = 25.4 Hz), 157.17 (d, J = 25.4 Hz), 148.2, 148.66, 139.94 (d, J = 234.3 Hz), 139.87 (d, J = 234.3 Hz), 121.75 (d, J = 34.2 Hz), 121.58 (d, J = 34.2 Hz), 87.97, 87.87, 85.20, 84.99, 70.75, 70.74, 70.27, 70.21, 33.04, 32.92, 28.78, 28.73, 26.51, 26.47, 23.76, 23.70. Figure 3. Micro-elemental analysis of MFU showing the percent by mass of the elements carbon, hydrogen and nitrogen in the structure of MFU compared to theoretical estimates. Figure 4. HPLC analysis of MFU showing a sharp peak at retention time of 1.85, performed by an independent laboratory using acetonitrile/water at a gradient of 20–90% with triethylammonium acetate (50 mM) additive. Figure 5. HRMS analysis of MFU. Supplement Table 1. Pyrimidine derivatives currently used as anticancer in clinical settings. Figure 6. NFKB taking at 1 sec (a), 2 secs (b), 3 secs (c) and 4 secs (d). Figure 7. FADD images taken at 1 sec (a), 6 secs (b), 12 secs (c) and 18 secs (d). Figure 8. B-actin images taken at 1 sec (a), 2 secs (b), 3 secs (c) and 4 secs (d). Figure 9. Images of BAX taken at 1 sec (a), 6 secs (b), 12 secs (c) and 18 secs (d). Figure 10. Caspase-9 images taken at 1 sec (a), 6 secs (b), 12 secs (c) and 18 secs (d). Figure 11. p53 images taken at 1 sec (a), 3 secs (b), 6 secs (c) and 9 secs (d). Figure 12. BCL-XL images taken at 1 sec (a), 3 secs (b), 6 secs (c) and 9 secs (d). Figure 13. In vitro studies comparing the efficiency of 5-FU and MFU. a) 2D culture of MiaPaca-2 cells, b) 2D culture of Panc-1 cells, c) 3D culture of Miapaca-2 cells and d) 3D culture of Panc-1 cells. [file 12885_2022_10449_MOESM1_ESM.docx]

**Supporting Figure 1**

Figure 1 ^1^H NMR analysis for MFU: ^1^H NMR (CDCl_3,_ 300 MHz): 7.36 (1H, d, *J_H-F_* = 6.0 Hz), 6.60 -6.67 (1H, m), 5.97 - 5.99 (1H, m), 4.32 (1H, td, *J* = 6.3, 8.4 Hz), 4.23 (1H, dt, *J* = 4.2, 8.7 Hz), 3.93 - 4.04 (2H, m), 2.44 - 2.54 (1H, m), 2.33 - 2.42 (2H, m), 2.20 - 2.28 (1H, m), 2.03 - 2.13 (2H, m), 1.86 - 1.96 (2H, m).

**Supporting Figure 2**

Figure 2 ^13^C NMR analysis for MFU: ^13^C NMR (CDCl_3_, 151 MHz) δ (a mixture of two rotamers): 157.28 (d, J = 25.4 Hz), 157.17 (d, J = 25.4 Hz), 148.2, 148.66, 139.94 (d, J = 234.3 Hz), 139.87 (d, J = 234.3 Hz), 121.75 (d, J = 34.2 Hz), 121.58 (d, J = 34.2 Hz), 87.97, 87.87, 85.20, 84.99, 70.75, 70.74, 70.27, 70.21, 33.04, 32.92, 28.78, 28.73, 26.51, 26.47, 23.76, 23.70.

**Supporting Figure 3**

Figure 3: Micro-elemental analysis of MFU showing the percent by mass of the elements carbon, hydrogen and nitrogen in the structure of MFU compared to theoretical estimates

**Supporting Figure 4**

Figure 4: HPLC analysis of MFU showing a sharp peak at retention time of 1.85, performed by an independent laboratory using acetonitrile/water at a gradient of 20-90% with triethylammonium acetate (50 mM) additive.


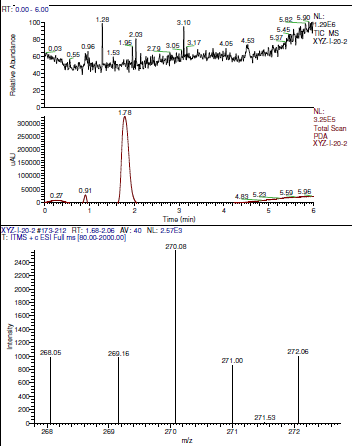


**Supporting Figure 5**

Figure 5: HRMS analysis of MFU

Supplement table 1: Pyrimidine derivatives currently used as anticancer in clinical settings

| **Derivative** | **Structure** | **Anti-cancer uses** |
| --- | --- | --- |
| 5-FU | 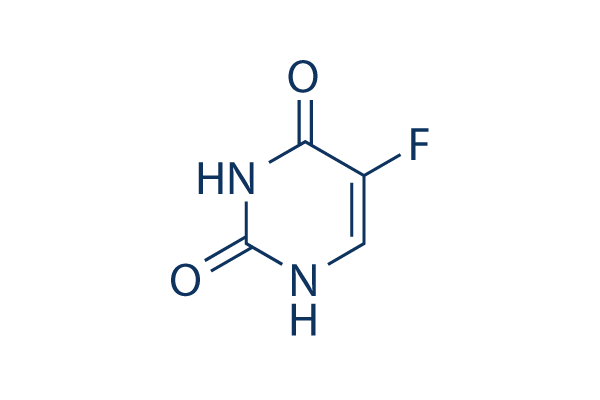 | Colorectal, breast and pancreatic cancers |
| Gemcitabine | 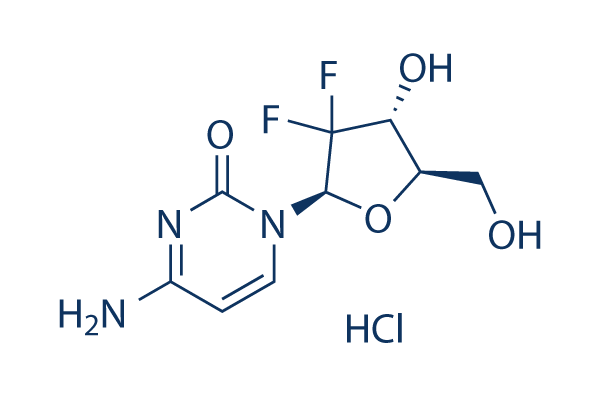 | Pancreatic, breast and bladder cancers |
| Capecitabine | 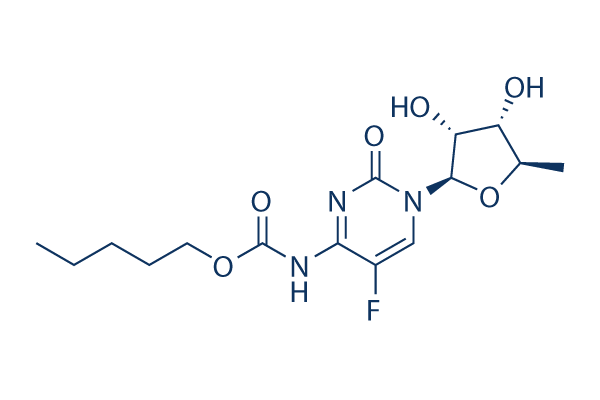 | Breast, advanced colon and rectal cancers |
| Tegafur | 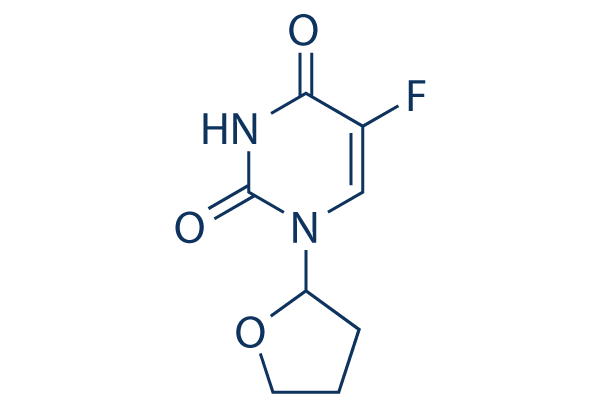 | Advanced gastric and colorectal cancers |

**Supporting Figure 6-12 Western Blot images (images in red borders are included in the manuscript) and images labelled a-d are captured at different time points**

1. **NFKB**


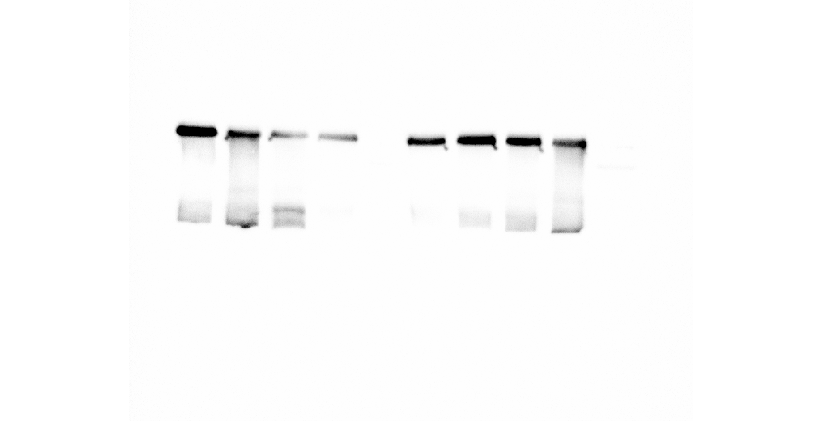


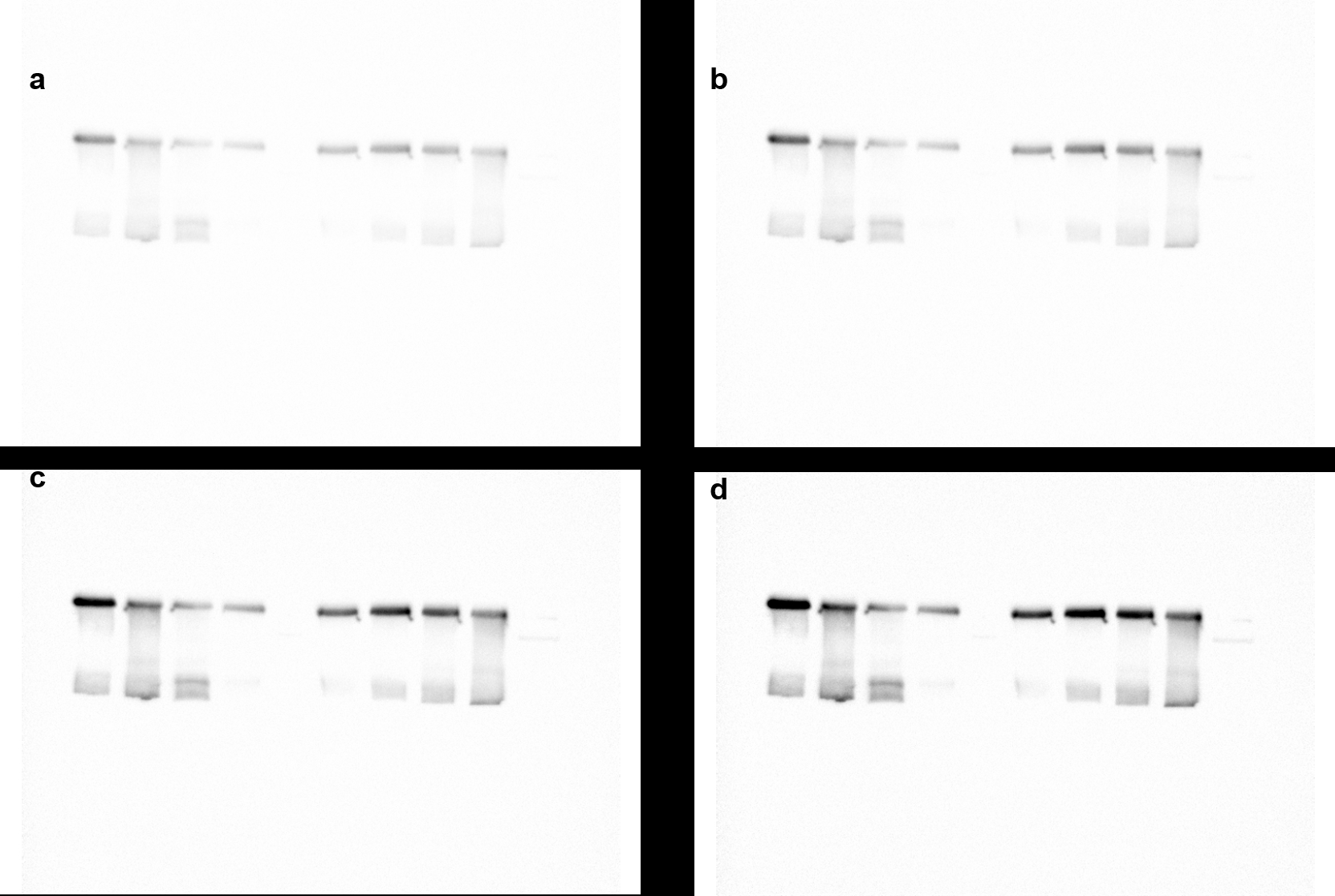


Figure 6: NFKB taking at 1 sec (a), 2 secs (b), 3 secs (c) and 4 secs (d)

1. **FADD**


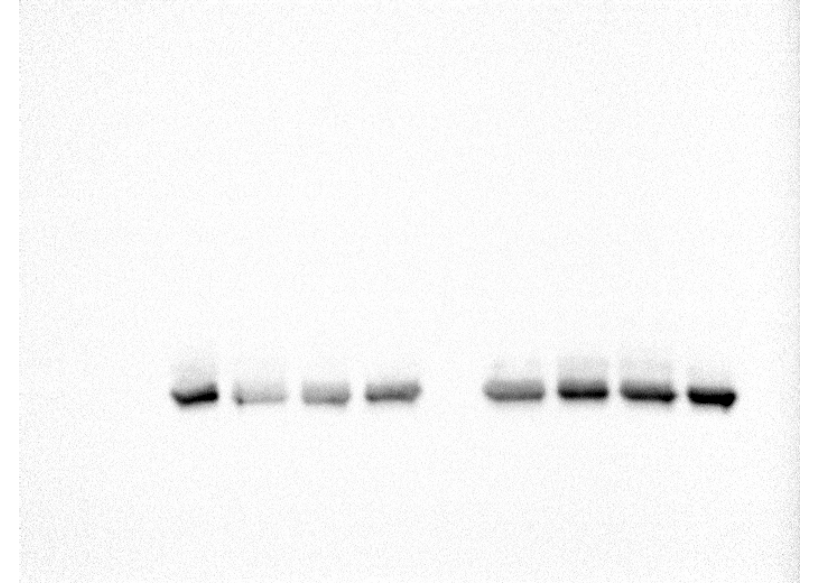


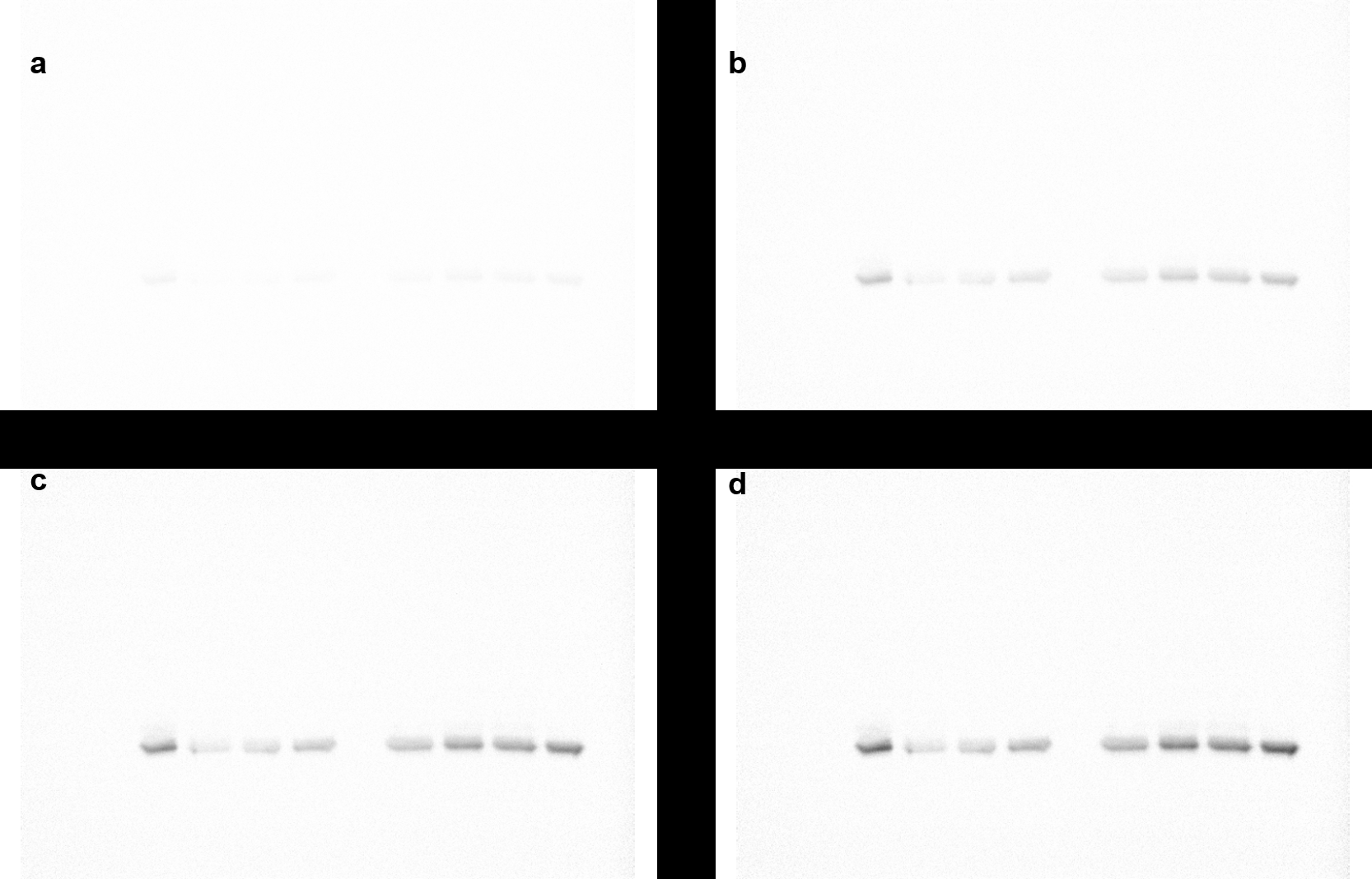


Figure 7: FADD images taken at 1 sec (a), 6 secs (b), 12 secs (c) and 18 secs (d)

1. **B-actin**


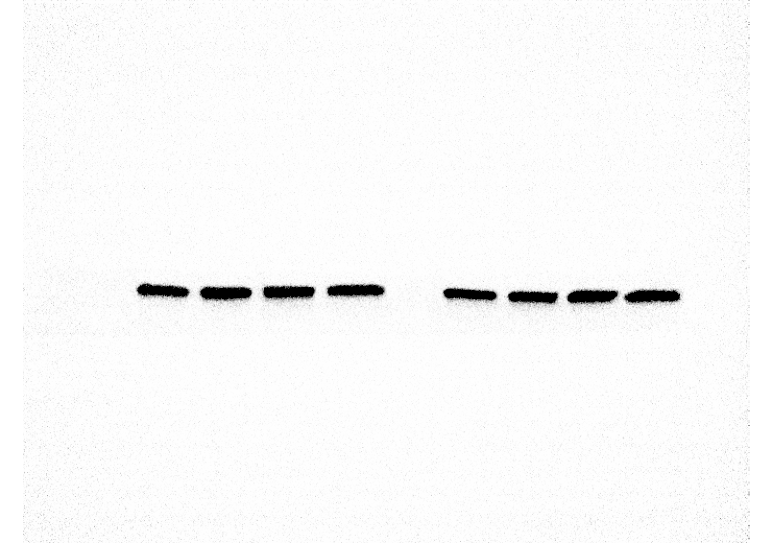


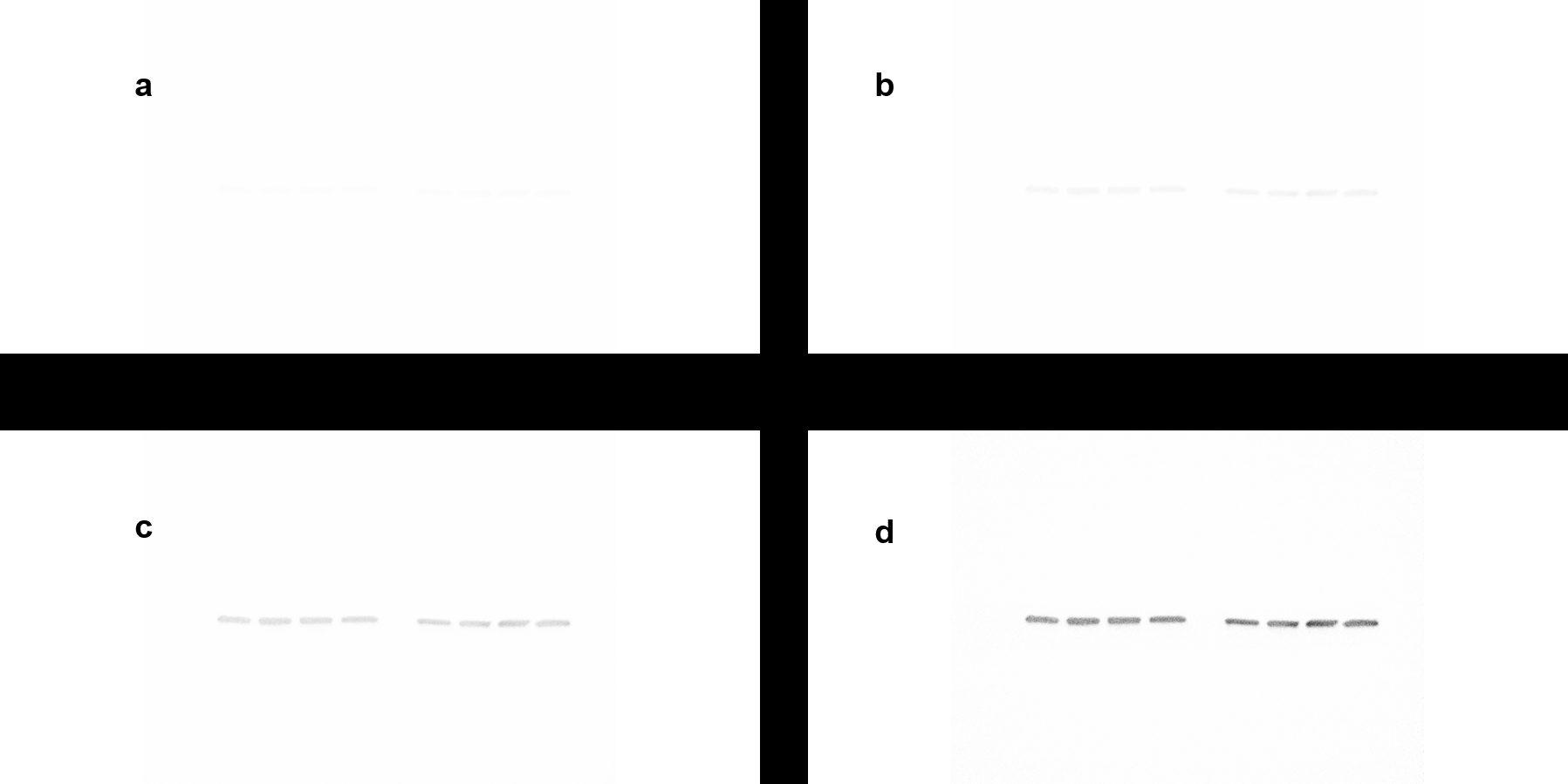


Figure 8: B-actin images taken at 1 sec (a), 2 secs (b), 3 secs (c) and 4 secs (d)

1. **BAX**


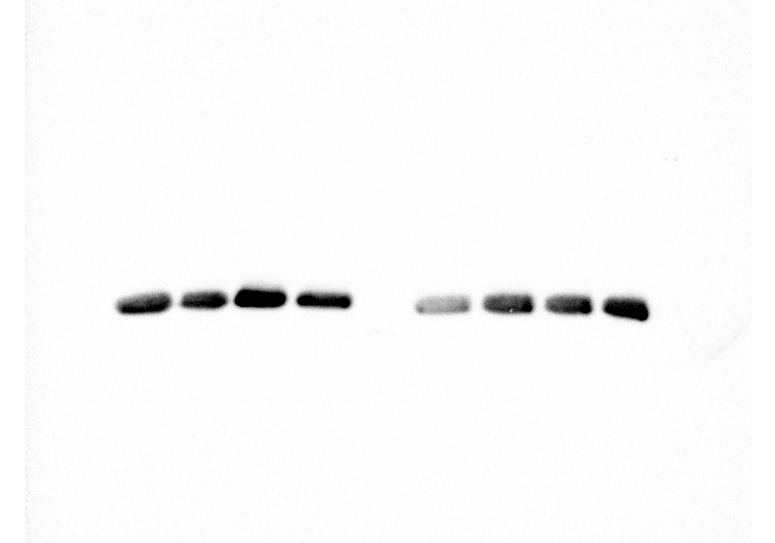


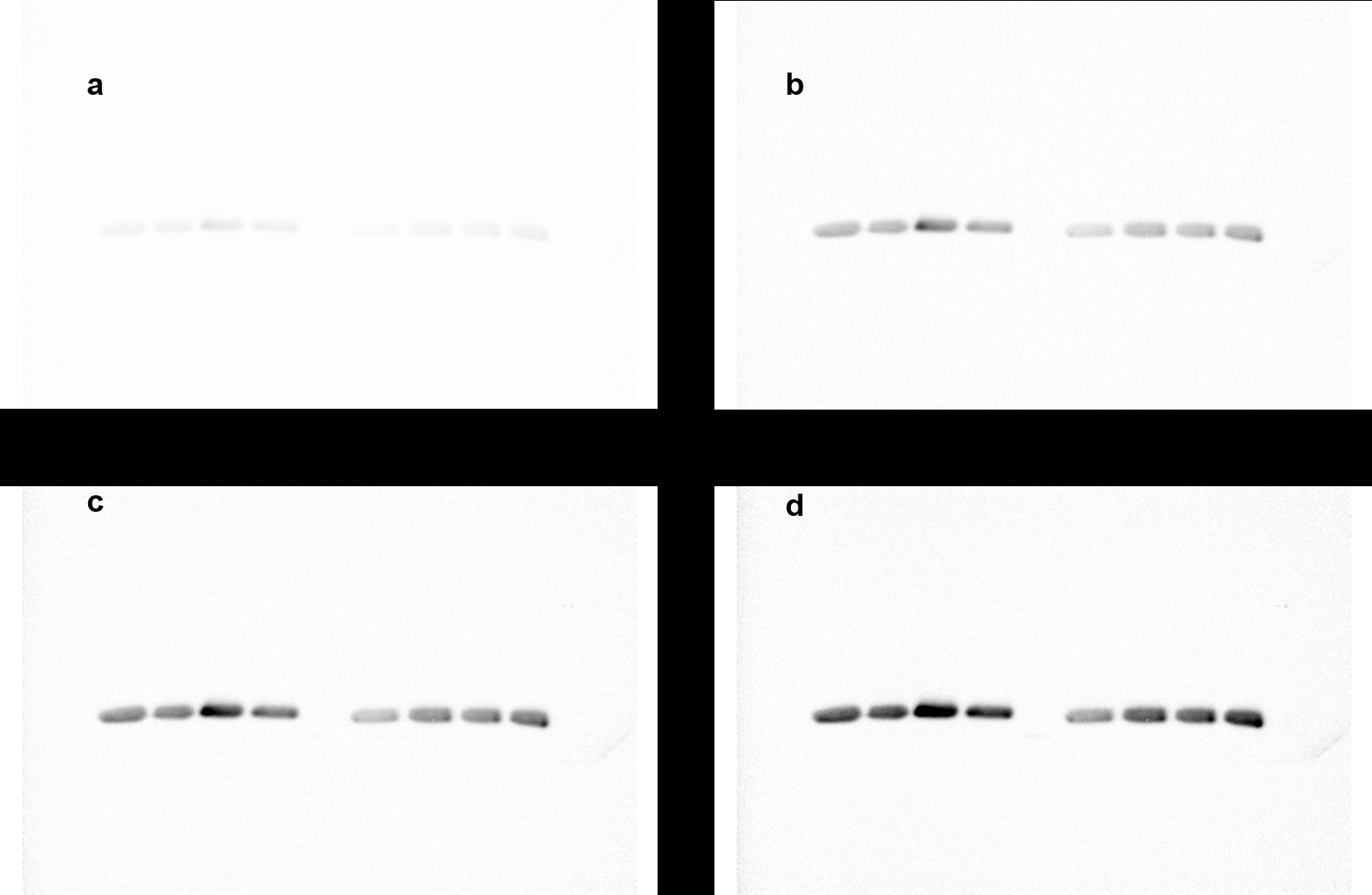


Figure 9: Images of BAX taken at 1 sec (a), 6 secs (b), 12 secs (c) and 18 secs (d)

1. **Caspase-9**


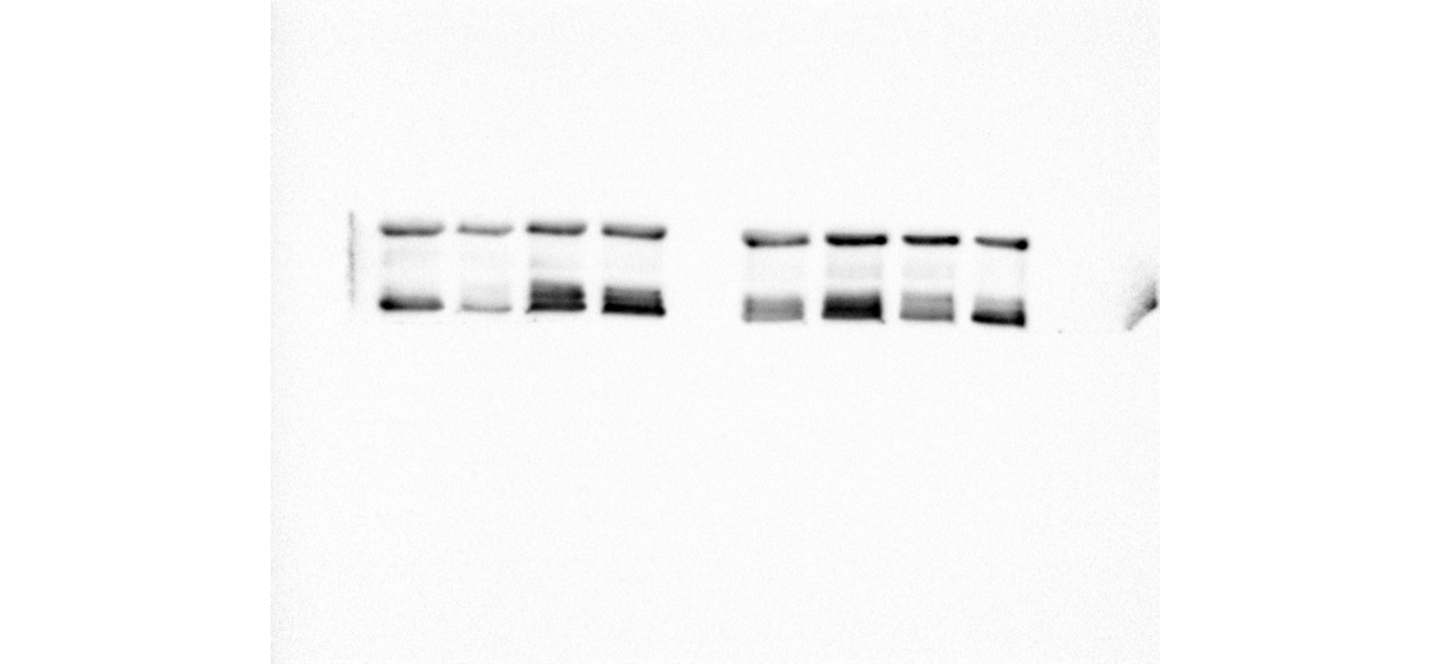


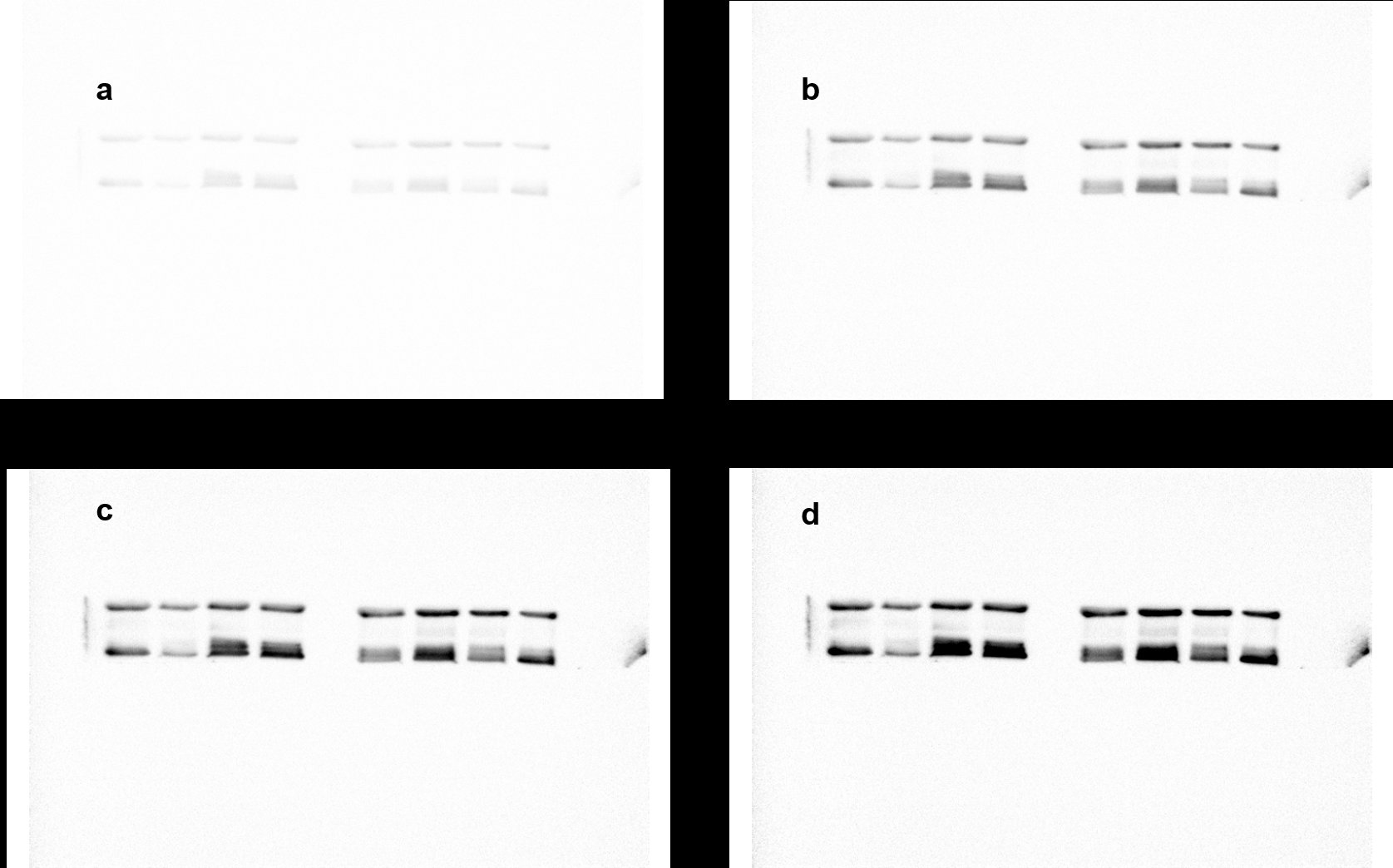


Figure 10: Caspase-9 images taken at 1 sec (a), 6 secs (b), 12 secs (c) and 18 secs (d)

1. **P-53**


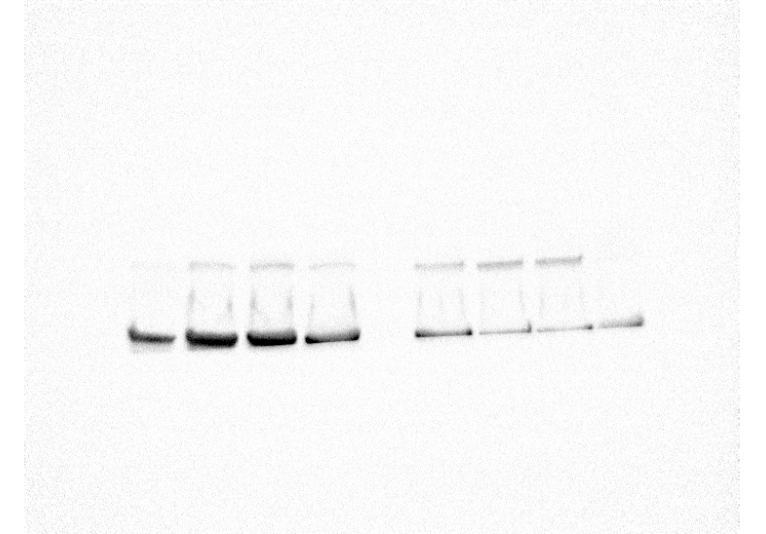


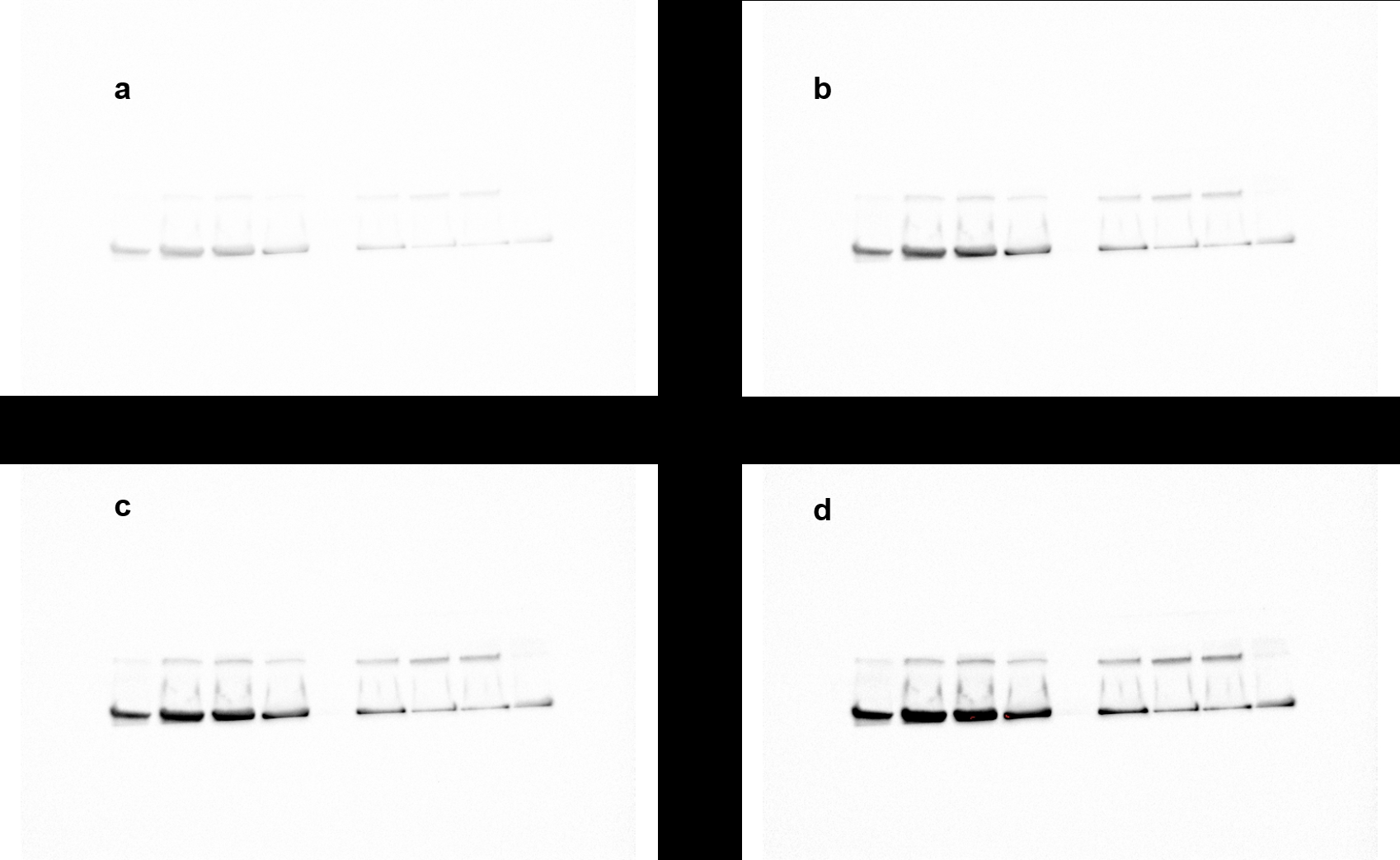


Figure 11: p53 images taken at 1 sec (a), 3 secs (b), 6 secs (c) and 9 secs (d)

1. **BCL-XL**


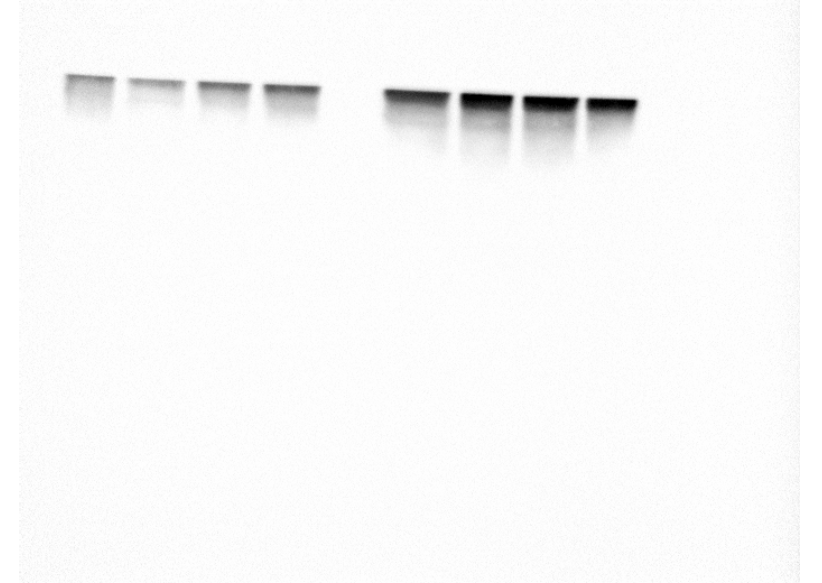


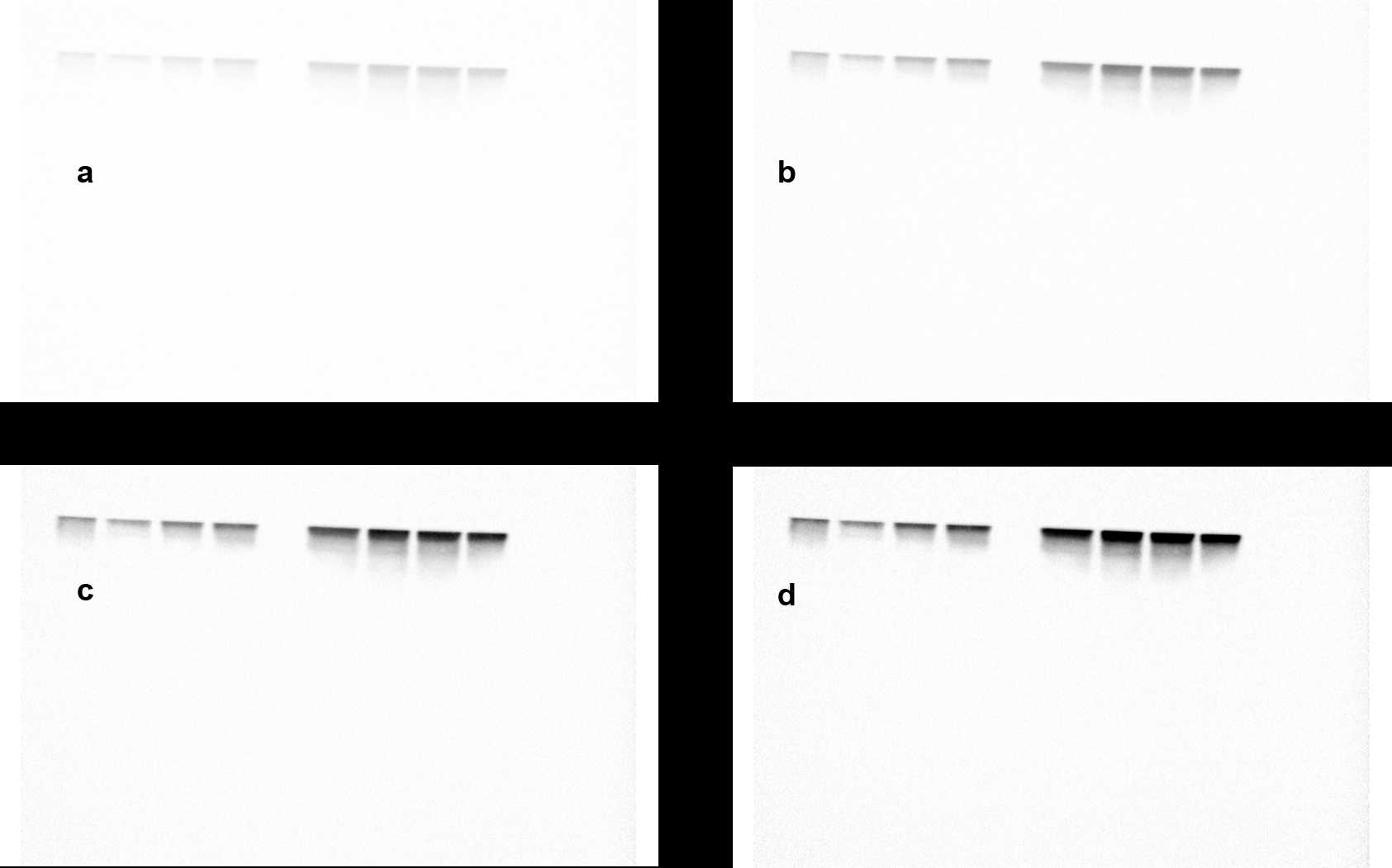


Figure 12: BCL-XL images taken at 1 sec (a), 3 secs (b), 6 secs (c) and 9 secs (d)

**Supplementary Figure 13**

**
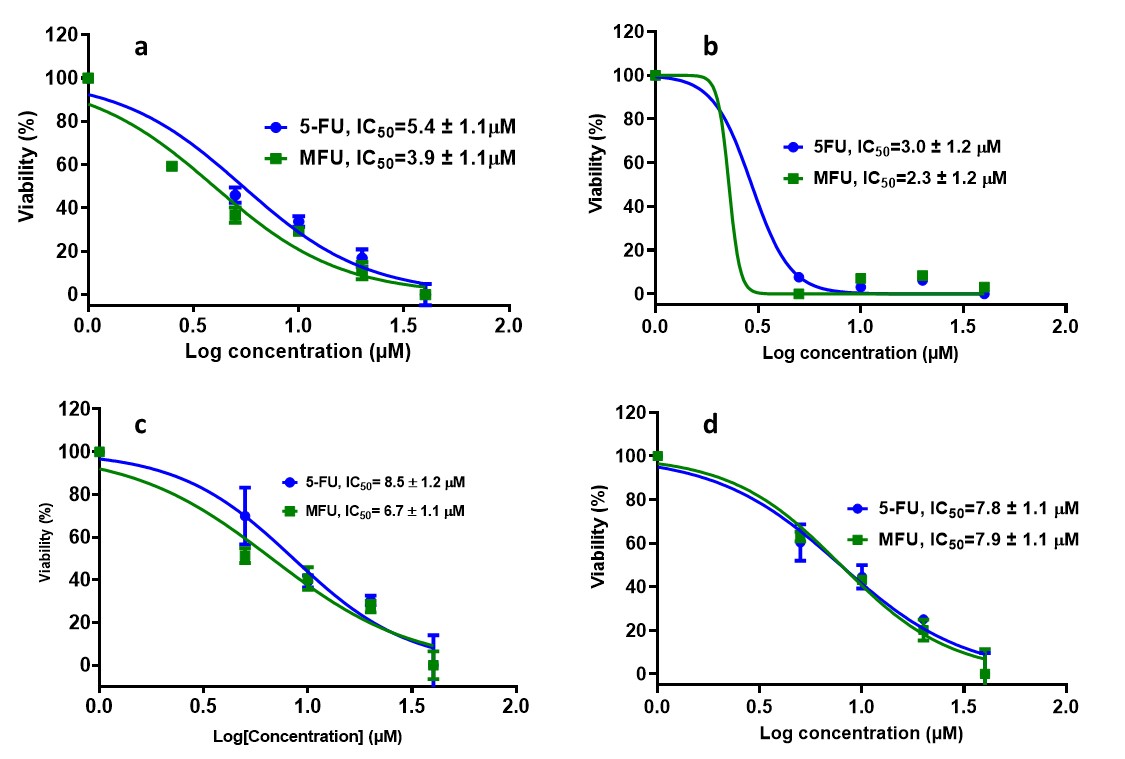
**

Figure 13: In vitro studies comparing the efficiency of 5-FU and MFU. a) 2D culture of MiaPaca-2 cells, b) 2D culture of Panc-1 cells, c) 3D culture of Miapaca-2 cells and d) 3D culture of Panc-1 cells.
